# The impact of the PEPFAR funding freeze on HIV deaths and infections: a mathematical modelling study of seven countries in sub-Saharan Africa

**DOI:** 10.1016/j.eclinm.2025.103233

**Published:** 2025-04-25

**Authors:** Jan A.C. Hontelez, Hannah Goymann, Yemane Berhane, Parinita Bhattacharjee, Jacob Bor, Sungai T. Chabata, Frances Cowan, Joshua Kimani, Justin Knox, Wezzie S. Lora, Cynthia Lungu, Jennifer Manne-Goehler, Joy Mauti, Mosa Moshabela, Rose M. Mpembeni, Mwanza Wa Mwanza, Thumbi Ndung'u, Evans Omondi, Sam Phiri, Mark Siedner, Frank C. Tanser, Sake J. de Vlas, Till W. Bärnighausen

**Affiliations:** a Department of Public Health, Erasmus MC, University Medical Center Rotterdam, Rotterdam, the Netherlands; b Heidelberg Institute of Global Health (HIGH), Medical Faculty and University Hospital, Heidelberg University, Germany; c Department of Epidemiology and Biostatistics, Addis Continental Institute of Public Health, Addis Ababa, Ethiopia; d Institute of Global Public Health, University of Manitoba, Winnipeg, Manitoba, Canada; e Partners for Health and Development in Africa, Nairobi, Kenya; f Department of Global Health and Epidemiology, School of Public Health, Boston University, Boston, MA, USA; g Centre for Sexual Health and HIV AIDS Research (CeSHHAR) Zimbabwe, Harare, Zimbabwe; h Department of International Public Health, Liverpool School of Tropical Medicine, Liverpool, UK; i Department of Psychiatry, Columbia University Irving Medical Center, New York, New York, USA; j HIV Center for Clinical and Behavioral Studies, NewYork State Psychiatric Institute and Columbia University, New York, New York, USA; k Department of Sociomedical Sciences, Columbia University Mailman School of Public Health, New York, New York, USA; l Public Health Group, Malawi Liverpool Wellcome Trust-Clinical Research Programme, Lilongwe, Malawi; m Center for Infectious Disease Research in Zambia (CIDRZ), Lusaka, Zambia; n Department of Pathology, Erasmus University Medical Center, Rotterdam, the Netherlands; o Division of Infectious Diseases, Brigham and Women's Hospital, Medical Practice Evaluation Center, Massachusetts General Hospital, Harvard Medical School, Boston, USA; p University of Cape Town, Cape Town, South Africa; q Muhimbili University of Health and Allied Sciences, School of Public Health and Social Sciences, Dar es Salaam, Tanzania; r Africa Health Research Institute (AHRI), Somkhele and Durban, South Africa; s HIV Pathogenesis Programme, The Doris Duke Medical Research Institute, University of KwaZulu-Natal, Durban, South Africa; t Ragon Institute of Mass General Brigham, Massachusetts Institute of Technology and Harvard University, Cambridge, Massachusetts, USA; u Division of Infection and Immunity, University College London, London, UK; v African Population and Health Research Center (APHRC), Nairobi, Kenya; w Institute of Mathematical Sciences, Strathmore University, Nairobi, Kenya; x Partners in Hope, Lilongwe, Malawi; y School of Global and Public Health, Kamuzu University of Health Sciences, Lilongwe, Malawi; z Department of Medicine, Harvard Medical School, Boston, MA, USA; aa South African Centre for Epidemiological Modelling and Analysis, Centre for Epidemic Response and Innovation, School for Data Science and Computational Thinking, Stellenbosch University, Stellenbosch, South Africa; bb Department of Global Health and Population, Harvard T.H. Chan School of Public Health, Harvard University, Boston, Massachusetts, USA

**Keywords:** HIV, PEPFAR, President's emergency plan for AIDS relief, Funding freeze, Executive order, HIV deaths, HIV incidence, HIV infections, Mathematical modeling, STDSIM, Africa, sub-Saharan Africa, Impact, ART, HIV treatment, Treatment interruption, Antiretroviral therapy, Model, Individual based model

## Abstract

**Background:**

On January 24, 2025, the United States government issued an executive order to freeze all foreign aid programs, including The President's Emergency Plan for AIDS Relief (PEPFAR), for 90 days. A limited waiver option became available, but its implementation remains incomplete. We estimated the impact of these policy changes on HIV deaths and new infections in seven sub-Saharan African (SSA) countries—Ethiopia, Kenya, Malawi, South Africa, Tanzania, Zambia, and Zimbabwe –, which together account for about half of all people living with HIV in SSA.

**Methods:**

We used STDSIM, an established individual-based simulation model, and previously published quantifications for the seven countries. We predicted changes in HIV deaths and new infections over the period 2025–2030 for four scenarios: (1) *Executive order*—*proportional*, where treatment disruption was proportional to the country-specific PEPFAR's share of total HIV funding; (2) *Executive order*—*realistic*, assuming near-total system collapse due to program dependencies; and (3–4) *Waiver* scenarios where treatment was resumed after 4 or after 8 weeks. Resumptions of programs accounted for delays due to organizational and logistical challenges.

**Findings:**

A 90-day funding freeze would result in 60 thousand [95% UI: 49–71 thousand] excess HIV deaths for the *Executive order*—*proportional* scenario. This number would increase to 74 thousand excess HIV deaths [95% UI: 63–89 thousand] for the *Executive order*—*realistic* scenario. Under a 4-week and 8-week waiver scenario, projected excess HIV deaths ranged between 21 thousand [95% UI: 15–28 thousand] and 28 thousand [95% UI: 22–36 thousand] respectively. Excess new infections ranged between 35 and 103 thousand for the different scenarios.

**Interpretation:**

The sudden cessation of PEPFAR funding likely results in tens of thousands of HIV deaths and new infections. These losses of life and health should compel the United States government to rapidly and fully re-instate one of the most successful health programs in history.

**Funding:**

None.

**Research in context**

**Evidence before this study**

On January 24, 2025, the United States State Department issued an executive order to freeze all foreign aid programs, including The President's Emergency Plan for AIDS Relief (PEPFAR), for 90 days. While a limited waiver option for PEPFAR became available, its implementation across programs remains incomplete. We searched PubMed for academic articles published in 2025 that assessed the impact of this funding freeze on HIV deaths and new infections in sub-Saharan Africa (SSA), in any language, using the search terms “pepfar” or “President's Emergency Plan for AIDS Relief” (search date: 10th of March 2025), and identified 10 articles relevant for our research question. Additionally, we searched for articles published by relevant newspapers, foundations and organizations. Despite the general agreement about the devastating short- and long-term impact of the PEPFAR funding freeze, by now, there are only two estimates of the consequential increase in HIV mortality and incidence in sub-Saharan Africa and globally. First, early estimates of *Tram* and colleagues showed that the 90-day funding freeze would result in over 100 thousand HIV-related deaths over one year, yet they did not consider time-lags in the impact of stopping treatment, delays in treatment resumption due to organizational and logistical challenges, did not incorporate HIV transmission effects, and did not consider different executive order and waiver scenarios. Second, *Gandhi* and colleagues estimated that a full retreat of PEPFAR from South Africa's HIV program could result in up to 565 thousand additional HIV infections by 2034 in South Africa and highlighted the impact of the additional economic burden, yet they did not evaluate the current 90-day funding freeze.

**Added value of this study**

We are the first to comprehensively predict the impact of the PEPFAR funding freeze in sub-Saharan Africa by accounting for time-lags in the impact of stopping treatment and for the delays in treatment resumption due to organizational and logistical challenges, by incorporating HIV transmission effects, and by considering different executive order and waiver scenarios. We used an established simulation model of HIV transmission and control to estimate the impact of the PEPFAR funding freeze on HIV incidence and mortality for seven countries in sub-Saharan Africa: Ethiopia, Kenya, Malawi, South Africa, Tanzania, Zambia, and Zimbabwe, which together account for about half of all people living with HIV in sub-Saharan Africa. We demonstrate that a combination of time delays in treatment resumption and increased new infections result in elevated mortality and incidence rates of up to five years beyond the initial funding freeze period. In total, we predict the total number of excess HIV deaths to reach about 60–74 thousand over the period 2025–2030 under different 90-day freeze scenarios, and between 20 and 28 thousand under different waiver scenarios, for the seven countries in our analysis. Excess new infections ranged between 35 and 103 thousand for the different scenarios.

**Implications of all the available evidence**

First and foremost, the devastating impacts identified in our study call for a rapidly and full re-instatement of PEPFAR, one of the most successful health programs in the history of public health. Second, global partnerships should rapidly mobilize to develop and deploy mitigation strategies to prepare for a partial or full retreat of the United States from the global HIV response. Third, local research and implementation programs should provide empirical evidence through measuring treatment defaults and HIV deaths in the coming months and years, and should work on implementing HIV treatment program innovations which can mitigate future, long-term funding reductions.

## Introduction

On January 24, 2025, four days after President Trump's second inauguration, the United States State Department sent out an executive order to freeze all US foreign aid funding, including the President's Emergency Plan for AIDS Relief (PEPFAR), for an initial period of 90 days.^1^ Over the past two decades, PEPFAR has been essential to the success of the rapid worldwide scale-up of antiretroviral therapy (ART) for HIV, especially in sub-Saharan Africa (SSA), the epicenter of the HIV pandemic.^2^ In little over 20 years, the number of people receiving life-saving ART in SSA has increased from nearly zero in the early 2000s to about 21 million in 2023.^3^ As a result, HIV/AIDS related mortality has dropped from around 2.2 million deaths in 2003 to about 390 thousand in 2023.^4^ Furthermore, as ART substantially reduces the infectiousness of those living with HIV, the scale-up has also contributed to the impressive decline in new HIV acquisitions, from 3·2 million in 2003 to about 640 thousand in 2023.^4^ By 2024, PEPFAR provided ART for 20·6 million people living with HIV and directly supported 342 thousand health workers.^5^

As of February 1, 2025, a limited waiver option was introduced, which offered to exempt PEPFAR programs that deliver life-saving HIV care and treatment and mother-to-child transmission prevention services from the funding freeze.^6^ Reports from African treatment programs, however, show that the possibility of obtaining a waiver has not yet resulted in treatment activities restarting in many cases, because of waiver application and implementation delays and organizational, administrative, and logistic constraints to restarting programs after they obtained a waiver.^7^^,^^8^ Local implementers are struggling to understand which funding streams and programs have been exempt through the waiver, and since the majority of USAID personnel has been put on leave,^9^ communication with PEPFAR is severely hampered.^10^ In addition, local programs have already dismissed health workers due to the immediate stop-order issued on January 24, and these are not easily to reinstate or replace.^11^ Finally, many USAID supported programs, including HIV programs, received termination notices on February 26th, further crippling HIV care and prevention.^12^ This situation will likely lead to substantial impacts for people living with HIV and the general population at risk of acquiring HIV.

In this study, we estimate these impacts. Mathematical models can bring to light and quantify these impacts in a better way than mere human intuition, because disruptions of HIV treatment programs will not immediately result in increased mortality risks for most individuals, as people can deplete their supplies over time and viral rebound takes a few weeks to occur.^13^ Therefore, those who have just started HIV treatment and suffered from advanced HIV disease prior to treatment initiation are at high risk of dying following treatment interruption. In addition, resumption of HIV program activities, either after a waiver or after the 90-day freeze, is unlikely to result in a rapid re-initiation of all those who defaulted treatment due to organizational and logistical challenges.

We applaud the early assessments by Tram et al., showing that abruptly stopping treatment for 90 days would result in over 100 thousand excess HIV deaths in SSA.^14^ Here, we take the crucial next step in this research by, firstly, accounting for time-lags in the impact of stopping treatment modelling for the delays in treatment resumption due to organizational and logistical challenges, secondly, incorporating HIV transmission effects and, thirdly, considering different executive order and waiver scenarios. We estimated the impact of these scenarios on HIV mortality and HIV incidence in seven PEPFAR priority countries in SSA^15^: Ethiopia, Kenya, Malawi, South Africa, Tanzania, Zambia, and Zimbabwe. Together, these countries account for about 15 out of the 26 million people living with HIV on the sub-continent, and recorded about 138 thousand HIV deaths, and 248 thousand new infections in 2023.^16^

## Methods

We used STDSIM, an established, individual-based simulation model of HIV transmission and control. The model captures country-specific transmission dynamics, and detailed supply- and demand-side dynamics of ART.^17^, ^18^, ^19^ For the current analysis, we adjusted the model to better reflect the impact of treatment interruptions on health (see schematic representation in Appendix Figure A1), and used quantifications from a previous study in which we estimated the impact of health systems constraints on the cost-effectiveness of ART in the ten biggest HIV epidemics in SSA.^18^

STDSIM was originally calibrated to the country-specific HIV prevalence and ART coverage from 1990 to 2014 using UNAIDS reported estimates. For each country, we selected a set of 40 unique parameter combinations, which reflect uncertainty in HIV transmission dynamics and ART uptake and were sampled from predefined ranges and accepted when model predictions fell within the fitting criteria, as described in Hontelez et al.^18^ We applied country specific ART guideline changes over time (i.e. to treatment eligibility at CD4 cell counts of ≤500 cells/mm^3^ and to treatment eligibility for everyone living with HIV, irrespective of CD4 cell count), and compared HIV prevalence and ART coverage over the period 1990–2023 to UNAIDS data.^16^ The patterns for 2015–2023 can thus be considered a validation of the model and its original quantifications. For seven out of the ten countries (Ethiopia, Kenya, Malawi, South Africa, Tanzania, Zambia, and Zimbabwe) the model predictions still closely matched UNAIDS HIV prevalence and ART coverage for the period 2015 and 2023. A detailed description of the model, including model validation against country-specific UNAIDS data, is given in the Appendix (Section 1).

We first determined the impact of full implementation of the executive order, i.e. a 90-day pause of HIV treatment programs, through two scenarios: in the *Executive order—proportional* scenario, the number of people on ART affected is proportional to the PEPFAR share in the total HIV budget of each country.^20^ The *Executive order—realistic* scenario reflects that PEPFAR funds critical components in the entire treatment delivery system, with its removal thus causing a near complete system collapse. For this scenario, we assumed that 90% of all people on ART are affected, except for Kenya and South Africa, where we chose 75% and 50% respectively. Here, the PEPFAR contributions are relatively smaller (i.e. 36% and 17% respectively of the total HIV budget). Second, we determined the impact of two *Waiver* scenarios, which differ in their duration until the program is resumed (after 4 weeks and 8 weeks respectively).

The scenarios were operationalized in the model as follows: during the period of the funding pause (90-days in the *Executive order* scenarios, and 4 and 8 weeks in the *Waiver* scenarios), those affected in either scenario randomly default treatment following a uniform distribution between 0 and 1 month after the implementation of the executive order, reflecting the fact that people may have stocks lasting up to 1 month. In addition, all new treatment initiations are immediately suspended during the pause. After the 90-day pause for both *Executive order* scenarios, we assumed a full resumption of PEPFAR funding. However, this does not immediately translate into pre-2025 treatment coverage levels, since the program would need to be fully restarted, staff needs to be re-hired, and people with HIV needing treatment need to be relocated and notified. We assumed that first the defaulted people will reinitiate treatment, taking on average 6 months. New initiations of treatment naïve individuals is resumed 6 months after resumption of funding.

After the 4 and 8 weeks pause in the *Waiver* scenario, resumption of PEPFAR will also not directly translate into a full recovery of treatment coverage. After the waiver comes into effect, we assumed treatment defaulting to stop, and defaulted people to re-initiate treatment at the same rate as for the *Executive order* scenarios (i.e. on average 6 months). New treatment initiations start at 25% of the pre-executive order level and remain there until the 90-day mark has passed, to reflect uncertainty in the sustainability of the program for both people with HIV and health care workers. After the 90-day deadline of the executive order has passed, treatment initiations are back at the pre-executive order level. An overview of the scenarios is given in Appendix Table A1, and the country-specific proportional PEPFAR shares in total HIV budgets are given in Appendix Table A2.

For each scenario, we predicted changes in HIV-related deaths and new infections compared to the counterfactual of continued unchanged funding and program operations, over the period 2025–2030. We ran 40 parameter combinations for each country and scenario, and the reported median and uncertainty intervals (UIs) in the manuscript reflect the median, 2·5th, and 97·5th percentiles of predictions across the 40 parameter combinations, reflecting uncertainty in the HIV transmission and ART uptake for each country.^18^ Model produced HIV mortality rates (per 1000 person years) and HIV incidence rates (per 100 person years), were converted to country-specific total numbers by multiplying the rates with population size estimates from the United Nations World Population Prospects 2024, using the “medium” variant for the years 2025–2030.^21^

### Role of the funding sources

None of the authors received funding for this work.

## Results

**Fig. 1:**
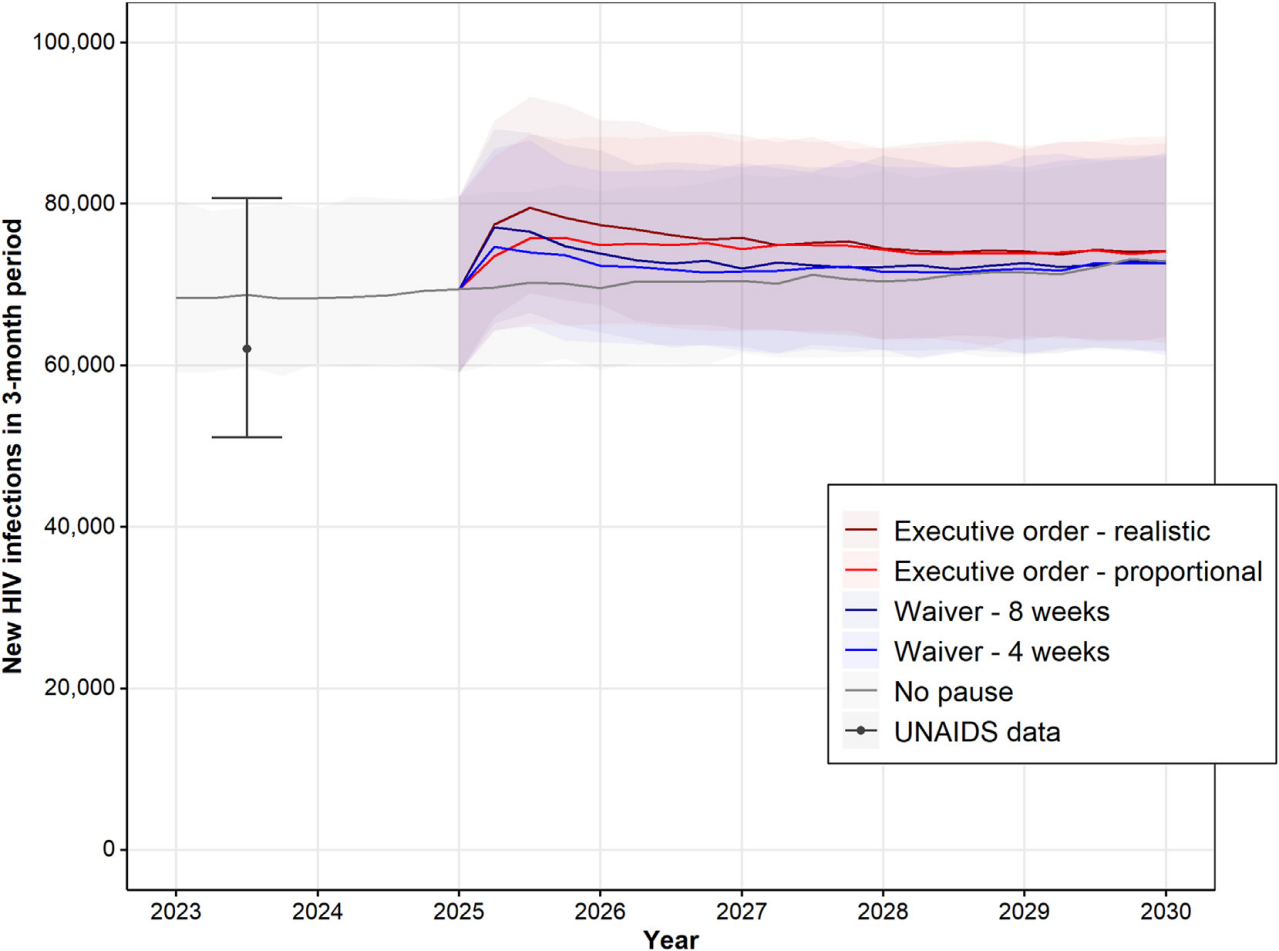
**Projected number of new HIV infections over the period 2023–2030 under different PEPFAR pause scenarios.** New infections are a combined total of Ethiopia, Kenya, Malawi, South Africa, Tanzania, Zambia, and Zimbabwe. Data point reflects combined UNAIDS data estimates for 2023. Lines represent median model predictions, shadings represent 95% uncertainty intervals (UIs), with borders of the shaded area representing the 2·5% and 97·5% quantiles of model predictions. A zoomed-in version of the Figure showing predicted number of new infections over the period 2025–2027 only is given in the Appendix (Figure A12).

Fig. 1 shows the predicted impact of the PEPFAR funding freeze scenarios on the absolute number of new infections for all seven countries combined. Country specific impacts on ART coverage and new infection trends are given in the Appendix (Figures A9 and A10, respectively). The *No pause* scenario (grey line in Fig. 1) predicts a stable number of new infections over the period 2023–2030, at around 70 thousand over a 3-month period. New infections over a 3-month period peak after 6 months since the start of the freeze for the *Executive order* scenarios (red lines in Fig. 1), at about 80 thousand (*Executive order—realistic*) and 75 thousand (*Executive order—proportional*). For the Waiver scenarios, new infections over a 3-month period peak after 3 months, at 77 thousand (*Waiver—8 weeks*) and 73 thousand (*Waiver—4 weeks*). New infections return back to the level of the no-freeze counterfactual after about 1–2 years in the *Waiver* scenarios, and 5 years in the *Executive order* scenarios. Our model predicts excess new infections to reach about 103 thousand [95% UI: 81–126 thousand] under the *Executive order—realistic* scenario, and about 81 thousand [95% UI: 64–98 thousand] under the *Executive order—proportional* scenario. Even under both *Waiver* scenarios, excess new infections reach 50 thousand [95% UI: 37–65 thousand] when PEPFAR is resumed after 8 weeks, and 35 thousand [95% UI: 19–50 thousand] when PEPFAR is resumed after 4 weeks (Appendix Table A3).

**Fig. 2:**
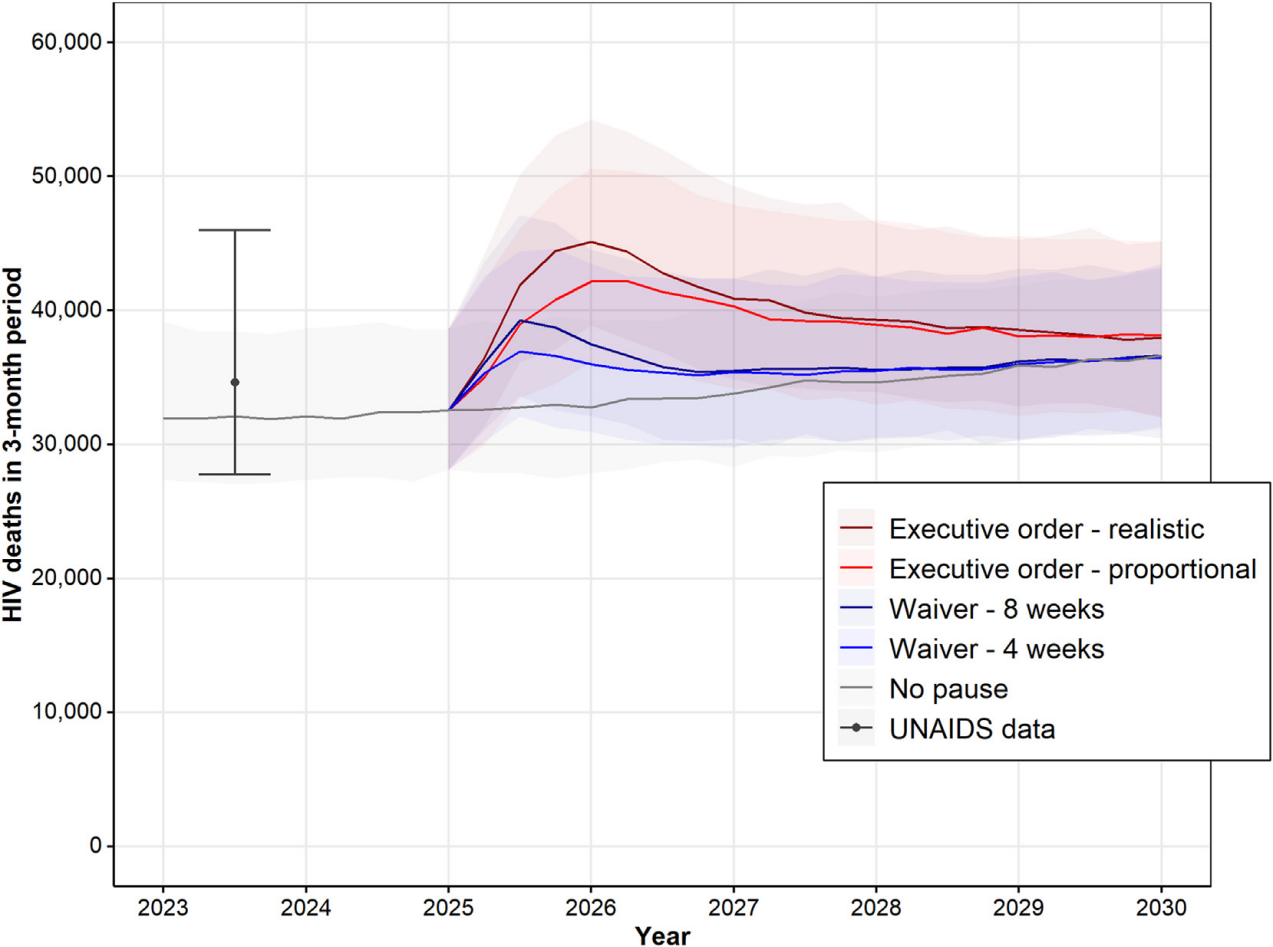
**Projected number of HIV deaths over the period 2023–2030 under different PEPFAR pause scenarios**. Results contain the following countries: Ethiopia, Kenya, Malawi, South Africa, Tanzania, Zambia, and Zimbabwe. Data point reflects combined UNAIDS data estimates for 2023. Lines represent median model predictions, shadings represent 95% uncertainty intervals (UIs), with borders of the shaded area representing the 2·5% and 97·5% quantiles of model predictions. A zoomed-in version of the Figure showing predicted number of HIV deaths over the period 2025–2027 only is given in the Appendix (Figure A13).

Fig. 2 shows that the PEPFAR freeze scenarios result in an immediate increase in new HIV deaths. In the *No pause* scenario (grey line in Fig. 2), number of HIV deaths every three months is predicted to slightly increase, from about 33 thousand in 2023 to about 36 thousand in 2030, due to a stable mortality rate and increasing population size. HIV deaths over a 3-month period peaks about 12 months after the start of the pause for the *Executive order* scenarios, at about 44 thousand (*Executive order—realistic*) and 42 thousand (*Executive order—proportional*). For the *Waiver* scenarios, the number of deaths over a 3-month period peaks 6 months after the start of the freeze, at about 39 thousand (*Waiver—8 weeks*) and 37 thousand (*Waiver—4 weeks*) HIV deaths respectively. It takes about 1·5–2 years for HIV coverage to return to pre-2025 levels for each country (see Appendix Figure A9), yet the absolute number of HIV deaths remains higher compared to the no pause baseline up to 5 years after the start of the pause in the *Executive order* scenarios, due to delayed treatment initiations and the increase in the number of new infections (Fig. 1). Even for the *Waiver* scenarios, it takes about 2.5 years for the absolute number of HIV deaths to return to the no-pause baseline level.

Our model predicts that a 90-day funding freeze would result in about 74 thousand [95% UI: 63–89 thousand] additional HIV deaths in the seven countries combined when assuming a near total collapse (*Executive order—realistic*) (Table 1). If treatment programs only collapse proportionally to the PEPFAR share, our model projects 60 thousand [95% UI: 49–71 thousand] excess HIV-related deaths (*Executive order—proportional*). Even under a *Waiver* scenario, excess HIV deaths were projected to range between 21 thousand [95% UI: 15–28 thousand] for a resumption after 4 weeks, and 28 thousand [95% UI: 22–36 thousand] for a resumption after 8 weeks. Out of the seven countries in our analysis, our model predicts most excess death to occur in South Africa, followed by Tanzania and Kenya (Table 1). While only about 1%–2% of all people living with HIV were within 1 year of starting treatment with a relatively low CD4 cell count (<350 CD4 cells/μL) for each country (Appendix Figure A14), this group accounted for between 10% and 20% of all excess HIV deaths across the countries (Appendix Figure A15). In addition, between 40% and 50% of excess HIV deaths originated from those who were treatment naïve at the start of the freeze (Appendix Figure A15).

**Table 1:** Excess number of HIV deaths (×1000) due to the PEPFAR funding freeze under different scenarios over the period 2025–2030.

Country	Executive order–realistic	Executive order–proportional	Waiver—8 weeks	Waiver—4 weeks
Ethiopia	2·2 [1·1; 2·9]	1·4 [0·6; 1·9]	0·2 [0·0; 0·5]	0·1 [0·0; 0·5]
Kenya	11·7 [9·8; 13·9]	8·4 [6·9; 10·1]	4·2 [3·0; 5·3]	2·8 [2·2; 3·7]
Malawi	6·9 [5·8; 7·7]	4·8 [3·9; 5·4]	2·5 [2·0; 3·0]	1·8 [1·1; 2·2]
South Africa	21·4 [19·2; 28·6]	17·7 [14·7; 23·2]	9·9 [8·0; 13·9]	7·7 [6·1; 11·4]
Tanzania	13·0 [11·4; 14·3]	12·0 [10·2; 12·9]	4·9 [3·3; 5·5]	3·4 [2·3; 4·2]
Zambia	10·7 [8·7; 11·8]	9·8 [8·2; 10·7]	3·7 [3·0; 4·4]	2·7 [2·1; 3·2]
Zimbabwe	8·1 [7·0; 9·4]	5·9 [4·7; 6·5]	2·8 [2·5; 3·2]	2·0 [1·6; 2·3]
Total	73·9 [63·0; 88·5]	60·0 [49·2; 70·7]	28·3 [21·7; 35·9]	20·6 [15·2; 27·6]

Estimates represent median model predictions, ranges represent 95% uncertainty intervals (UIs), i.e. the 2·5% and 97·5% quantiles of model predictions.

## Discussion

We show that the PEPFAR funding freeze would likely result in about 60–74 thousand additional HIV deaths in Ethiopia, Kenya, Malawi, South Africa, Tanzania, Zambia, and Zimbabwe combined, and that the effects extend far beyond the 90-day period due to delayed treatment initiations and increased transmission. Even under the most conservative waiver scenario, total excess HIV deaths reach over 20 thousand. Those most at risk for excess death are people who are treatment naïve, or within 1 year since treatment initiation with CD4 cell counts <350 cells/μL. In addition to these mortality burdens, we estimate that the PEPFAR funding freeze will cause between 35 thousand and 103 thousand new HIV infections in the seven countries combined.

Our study focused on seven countries in SSA, which cover about half of all people living with HIV on the sub-continent. Given that PEPFAR plays a key role in nearly all treatment programs across SSA,^5^ and generally funds critical components in the treatment delivery system in most countries (i.e. the *Executive order—realistic* scenario), the expected impact of the freeze scenarios may well reach 150 thousand excess HIV deaths and 200 thousand excess new infections for SSA as a whole. We only model impacts of the initial 90-day PEPAR freeze, and in this short time horizon, this scenario of collapsing HIV treatment programs is indeed realistic, even in countries such as South Africa, where PEPFAR only funds a small fraction of total HIV treatment programmatic costs. It is difficult to replace the funding for, or re-arrange operationally, individual critical components—such as procurement, inventory management, supply chain management, scheduling and quality control—without substantial lead time for government processes and operational preparation. Our estimates are higher than those provided by Tram et al. (about 100 thousand excess HIV deaths),^14^ likely due to the incorporated transmission effects in our study, as well as the delays in treatment resumption after the 90-day freeze due to organizational and logistical challenges.

We have estimated the impacts of the initial, short-term PEPFAR funding freeze and the subsequent waiver program. In our estimations, we assumed complete resumption of funding following the freeze. However, there is a real possibility that PEPFAR funding will remain substantially reduced, or completely stopped, after the 90-day review period. This policy scenario would likely imply that the US will also substantially reduce its share of funding for *the Global Fund to Fight AIDS, Malaria and Tuberculosis* (the *Global Fund*), another important institution and funding mechanism for HIV treatment and prevention. Currently, the US contributes about one third of the total *Global Fund* budget. Loss of this funding would likely induce further avoidable deaths and new HIV acquisitions. It is obvious that such possible policy scenarios would have even more detrimental impacts, as recently estimated by Gandhi et al. for South Africa,^22^ and Brink et al. for all low- and middle income countries.^23^ The modelling estimations from Ghandi et al. and Brink et al. are also aligned with the recent work from Walimbwa et al., which reports the immediate and detrimental impacts of the PEPFAR freeze and the subsequent waiver implementation on an HIV program and population health in Kenya.^24^ It is imperative that the international community starts to work on mitigation strategies in the event of a full retreat of the United States from the global health agenda, such as mobilizing alternative funding and developing guidance on optimal resource allocation and service delivery models within constrained systems.^25^^,^^26^

Our study focused only on the impact of the PEPFAR freeze on HIV treatment programs, as disruptions in these programs most immediately and directly endanger people's lives. Treatment saves the lives of people living with HIV and protects the lives of people not living with HIV, because successfully treated individuals do not transmit HIV to their sexual partners. Other PEPFAR-funded prevention programs, however, are an important complement to treatment-as-prevention. These include HIV pre-exposure prophylaxis,^27^^,^^28^ voluntary medical male circumcision (VMMC), condom distribution, targeted combination prevention programs for key populations,^29^ and programs for the prevention of mother-to-child transmission of HIV.^30^ Disruptions in these programs will further increase the number of new infections beyond our estimates and, over the long run, also the number of HIV deaths. Prevention programs supported by PEPFAR were not eligible for the limited waiver exempting funding from the 90-day freeze. They are therefore affected by the 90-day freeze, regardless of the scenarios in our analysis. While our model allows for simulating the impacts of prevention programs, our original quantifications were primarily developed to assess HIV treatment guidelines, and we lacked country-specific information on current PEPFAR funding levels and coverage for the different prevention programs.

Our model currently does not feature specific impacts of the PEPFAR freeze on programs for and the health of key populations. This is an important limitation of our work, which future studies should address. Key populations are disproportionally affected in the HIV pandemic, both because of their high vulnerability to infection and often their socioeconomic disadvantages impeding access to treatment and prevention services. Tailored, people-centered programs have been critical in ensuring that key populations have access to HIV prevention, testing, and treatment services, and the termination of these programs will specifically endanger the lives and livelihoods of these most vulnerable people, who we know have difficulty accessing general population services.

It is also important to note that our estimates do not capture the effects of increased development of HIV resistance due to declining blood drug levels following treatment interruption. Resistance development is a major risk of treatment interruption and irregular and incomplete treatment adherence, which the PEPFAR funding freeze is likely to have induced. Future empirical work needs to quantify the effects of the funding freeze on resistance and the resulting long-term consequences for the HIV treatment response. In the long run, our ability to treat and prevent HIV may diminish, as drug acquired and transmitted HIV drug resistance increases.^31^

Next to understanding the impact of the PEPFAR funding freeze scenarios on HIV deaths and new infections through modelling, it will be essential that local research and implementation programs provide empirical evidence through measuring treatment defaults and deaths in the coming months and years. In addition, local and global partners should work on implementing HIV treatment program innovations, which can mitigate future, long-term funding reductions. One major opportunity is task-shifting of HIV treatment from nurses in primary care clinics to community health workers who visit the homes of people on HIV treatment, delivering antiretroviral medicines, screening for side effects, and supporting long-term retention in care and treatment adherence.^32^ Finally, the HIV community should work closely with health policy makers in investigating and establishing approaches to integrate the largely disease-specific HIV treatment programs supported by PEPFAR into the general health system. In such integrations, it will be important to address stigma and discrimination faced by people living with HIV and key populations in order to maintain the high levels of effectiveness and efficiency that HIV treatment programs have achieved through their dedicated focus on supporting the treatment of a single disease, which has led to learning effects and economies of scale, while avoiding diseconomies of scope that could result following integration of HIV treatment programs into the general health system.

It is important to note that we only provide estimations of the real-world impacts of the PEPFAR funding freeze. The precise operationalization of pause and reinstatement scenarios in real world settings is uncertain.^33^ First, we assumed equal probabilities of defaulting and equal rates of re-initiating treatment regardless of disease stage, yet some triage strategies to ensure that those in most need are treated first may be put in place, potentially softening some of the blow. Nevertheless, such strategies require resources and guidelines to be operationalized effectively, none of which are currently in place. Second, we assumed all people receiving ART to have supplies between 0 and 1 month, yet many countries have implemented different forms of differentiated care, in which multi-month supplies are given to those who are stable and suppressed on ART. Nevertheless, most excess mortality in our model comes from those who are generally not eligible for multi-month supplies, i.e. those who are treatment naïve or on treatment for less than 1 year since the start of the freeze. Third, a more rapid reinstatement of programs after funding is resumed may further dampen some of the impact predicted in our models. Fourth, the Executive order—proportional scenario assumes treatment programs to be reduced proportionally to the PEPFAR share in the total HIV budgets for each country. While it is difficult to fully separate funding streams for treatment and prevention, especially concerning service delivery elements, estimated PEPFAR shares that directly fund treatment and care are close to the estimated total budget shares for each country (see additional column in Appendix Table A2).

We conclude that the sudden cessation of PEPFAR funding has immediate and far-reaching impact, likely resulting in tens of thousands of excess HIV deaths and new infections for the seven PEPFAR priority countries in our study. For the continent as a whole, the number of excess HIV deaths will be substantially higher because our study only covers about have of the total population living with HIV in Africa. The losses of life and health that we show should compel the United States government to rapidly and fully re-instate PEPFAR, one of the most successful health programs in the history of public health. Global partnerships should rapidly mobilize to develop and deploy mitigation strategies to prepare for a partial or full retreat of the United States from the global HIV response.

## Contributors

JACH, SJdV and TWB conceptualized and designed the study, JACH, HG, SJdV, and TWB performed analysis and interpretation, JACH wrote the first draft of the manuscript, all authors contributed to writing and editing the final version of the manuscript. JACH, SJdV, and TWB have accessed and verified the data, and were responsible for the decision to submit the paper.

## Data sharing statement

All underlying models and code will be made available upon reasonable request with the corresponding author.

## Declaration of interests

TWB received grants from the following institutions for the Heidelberg Institute of Global Health (HIGH): Horizon Europe, Horizon 2020, US National Institutes of Health, Wellcome, German National Research Foundation, German Ministry of Education and Research, Bill & Melinda Gates Foundation, Fleming Fund, UNAIDS, Health + Life Alliance Heidelberg-Mannheim, Alexander von Humboldt Foundation, International Vaccine Institute, Else Kröner Fresenius Foundation, League of European Research Universities (LERU), German Corporation of International Cooperation (GIZ), Volkswagen Foundation, German Development Bank (KfW), African Academy of Sciences and European and Developing Countries Clinical Trials Partnership. TWB received support for attending meetings from WHO, Disease Control Priorities Project 4, Peking Union Medical College (PUMC), Baden-Württemberg Foundation and Africa Health Research Institute (AHRI). All payments were made to the institution. TWB is the Chair of the International Scientific Advisory Board of the EU Horizon grant “HIGH Horizons—Heat Indicators for Global Health Monitoring, Early Warning Systems and health facility interventions for pregnant and postpartum women, infants and young children and health workers”, he is a member of the Scientific Advisory Board of the Leibniz Research Network INFECTIONS (LFV) and a member of the Virchow Foundation in Berlin. All services are unpaid. TWB works as the Editor in Chief for PLOS Medicine. FC holds the following grants: Wellcome Trust [Grant number:214280/Z/18/Z], Gates Foundation INV 066092 and NIMH R34 MH129220-01. Payments were made to his institution. JMG received grants from Gilead, Viiv, National Institutes of Health and WHO. All other authors declared no interests.
