# Supplementary material for: The impact of the PEPFAR funding freeze on HIV deaths and infections: a mathematical modelling study of seven countries in sub-Saharan Africa
Source: eClinicalMedicine. 2025 Apr 25;83:103233. doi: 10.1016/j.eclinm.2025.103233 (PMC12230335; doi:10.1016/j.eclinm.2025.103233)
Supplement: Appendix [file mmc1.pdf]

APPENDIX TO MANUSCRIPT

The impact of the PEPFAR funding freeze on HIV deaths and infections: a mathematical modelling study of seven countries in sub-Saharan Africa

Contents

1. Model description and quantification 3

1.1 HIV natural history and ART 3

Figure A1. Schematic representation of stages in adjusted HIV natural history and ART processes in STDSIM. 5

Figure A2. HIV mortality rate by CD4 cell count in untreated HIV, model compared to data. 6

Figure A3. Cumulative distribution of CD4 cell counts after 1 year of antiretroviral therapy, model compared to data. 6

Figure A4. Proportion never failing treatment (defined as experiencing a decline in CD4 cell counts while on ART) by time on treatment, model versus data. 7

Figure A5. Mortality by time on ART and CD4 cell count at treatment initiation, model versus data. 7

1.2 Country quantifications 8

Figure A6. Modelled versus UNAIDS reported HIV prevalence in people aged 15 years or older over the period 1990 to 2023. 9

Figure A7. Modelled versus UNAIDS reported ART coverage in people aged 15 years or older living with HIV over the period 1990 to 2023. 10

Figure A8. Modelled versus UNAIDS reported HIV related mortality in people aged 15 years or older over the period 1990 to 2023. 11

2. Funding freeze scenarios 12

Table A1. Overview of *Executive order* and *Waiver* scenarios in the model. 12

Table A2. Overview of PEPFAR shares in total HIV spending in each country, for 2022. 13

3. Country specific outcomes 14

Figure A9. Predicted impact of PEPFAR funding pause scenarios on ART treatment coverage in seven countries, 2023 to 2030. 15

Figure A10. Predicted impact of PEPFAR funding pause scenarios on new HIV infections in seven countries, 2023 to 2030. 16

Figure A11. Predicted impact of PEPFAR funding pause scenarios on HIV deaths in seven countries, 2023 to 2030. 17

Figure A12. Projected number of new HIV infections over the period 2025 to 2027 under different PEPFAR pause scenarios 18

Table A3. Excess number of new HIV infections (x 1,000) due to the PEPFAR funding freeze under different scenarios..........19

Figure A13. Projected number of HIV deaths over the period 2025 to 2027 under different PEPFAR pause scenarios. 20

Figure A14. Distribution of people living with HIV across ART treatment categories before the freeze. 21

Figure A15. Distribution of excess deaths across ART treatment categories at the start of the freeze for the *Executive order – realistic* scenario..........22

4. References..........23

1. Model description and quantification

We used STDSIM, a stochastic microsimulation model of the transmission and control of HIV and other sexually transmitted infections (STIs).[1–10] The model simulates the life course of individuals in a dynamic network of heterosexual contacts. Events like partnership formation, acquisition of infection, or seeking care are the result of random processes, determined by probability distributions. Therefore, the results of the model are subject to stochastic variation. The model consists of four modules: demography, sexual behaviour, transmission and natural history, and interventions. The *demography* module implements the processes of birth, death, and migration. Processes governing initiation and dissolution of sexual relationships, mixing according to age preference, sexual contacts within relationships, and sexual contacts between clients and sex workers are defined in the *sexual behaviour* module. In the *transmission and natural history* module, transmission probabilities per sexual contact are specified for HIV and four other STIs that act as cofactors: chlamydia, gonorrhoea, syphilis, and chancroid. Finally, the *interventions* module specifies the timing and effectiveness of control measures in curbing transmission or enhancing survival.

1.1 HIV natural history and ART

For the current analysis, we used a previously published version of the model that was used for country-specific calculations on health system constraints in ART provision.[10] In the model (see Figure A1 for a schematic), natural history of HIV is described by means of six consecutive stages, with exponentially distributed durations: i.e. early infection (average of 10 weeks), two stages of asymptomatic infection (average of 125 weeks, or 2·4 years, each), two stages of symptomatic infection (average of 120 weeks and 80 weeks, or 2·3 years and 1·5 years respectively), and AIDS (average of 40 weeks, or 0·8 years). Thus, the total average survival of an individual with untreated HIV until dying from AIDS is about 10 years. Each individual is assigned an HIV-negative CD4 cell count, drawn from a log-normal distribution calibrated against data, and CD4 cell counts decline by 25% during the acute stage, and linearly during the course of the other stages to 0·5% after 40 weeks in the AIDS stage (red line in top row of Figure A1).[5,9,10] ART treatment uptake in the model is the result of two processes. The first represents an individual's demand for ART as a function of disease stage, while the second describes the health systems capacity to meet the population's need. Rates of seeking care (α_i) are assumed to increase during disease progression, as HIV-infected people experiencing symptoms are more likely to seek care.[9,10]

Each untreated HIV stage has an ART-stage-equivalent to which individuals are moved to upon treatment initiation. In the original version of the model, people continued to progress to subsequent stages, be it at a much slower rate.[10] While this mechanism was adequate for simulating the population impact of ART, it is not able to accurately reproduce the fact that, in the event of sudden treatment interruptions, especially those who have recently started ART with relatively low CD4 cell counts would be at risk of dying, whereas those long on ART have an improved condition and higher CD4 cell counts. We therefore adjusted the HIV natural history and ART in the model in two ways to better capture HIV mortality processes when ART is interrupted: 1) people now experience an increased risk of HIV-related death during the last three stages, rather than only at the end of the AIDS stage, to better reflect that mortality rates are already higher before the onset of AIDS (μ_1 through μ_{AIDS});[11] and 2) people on ART now experience health recovery through a regression back to stages with higher CD4 cell counts over time

(middle row in Figure A1), yet can experience treatment failure in which disease progresses again (bottom row in Figure A1). We chose stage specific HIV mortality rates (μ_1 through μ_{AIDS}) and stage specific transitions rates (dashed arrows) so that the overall average duration of each stage for untreated HIV – as well as treatment failure – still match the original durations (120 weeks, 80 weeks, and 40 weeks) and that CD4 count specific HIV mortality rates reproduced data on the 6-month survival probabilities of people with HIV, by CD4 cell count, from Badri *et al* (Figure A2).[11]

Health seeking rates by disease stage (α_1 through α_5) and dropout rate (β) were maintained from the original model version, reflecting data on CD4 cell count at first test and proportion lost to follow-up from the treatment program at the African Health Research Institute (AHRI).[10,12] After treatment initiation, people now regress back through the stages at a rate of *q* times the untreated rates. A value for *q* was chosen so that the model matched data of CD4 cell count recovery after 1 year on treatment from the AHRI treatment program.[12] With a value of 0·5, the model closely reproduces the data, except for very low CD4 cell counts, i.e. below 20 cells/mm^3 (Figure A3). We further assumed that people on successful treatment in the last three stages of HIV still experience a higher rate of dying, yet reduced, as reflected by a multiplier *r* compared to untreated HIV. Furthermore, those on successful treatment experience a treatment failure rate of γ, after which they move to the bottom row in Figure A1 and start progressing through the stages again, similar to untreated HIV. A value for γ = 0·115/year was chosen so that the proportion ever experiencing failure by time on ART matched data from the AHRI treatment program (see Figure A4). Finally, people failing treatment can be re-suppressed at a rate δ, and a combination of values for *r* = 0·5 and δ = 0·55/year was chosen so that mortality by time on ART and CD4 cell count at treatment initiation matched data from the AHRI treatment program (see Figure A5). Values for parameters *r*, *q*, γ, and δ were chosen in combination, and we assumed the same background (i.e. non-HIV related) age-specific mortality as in the original ART calibrations.[10]

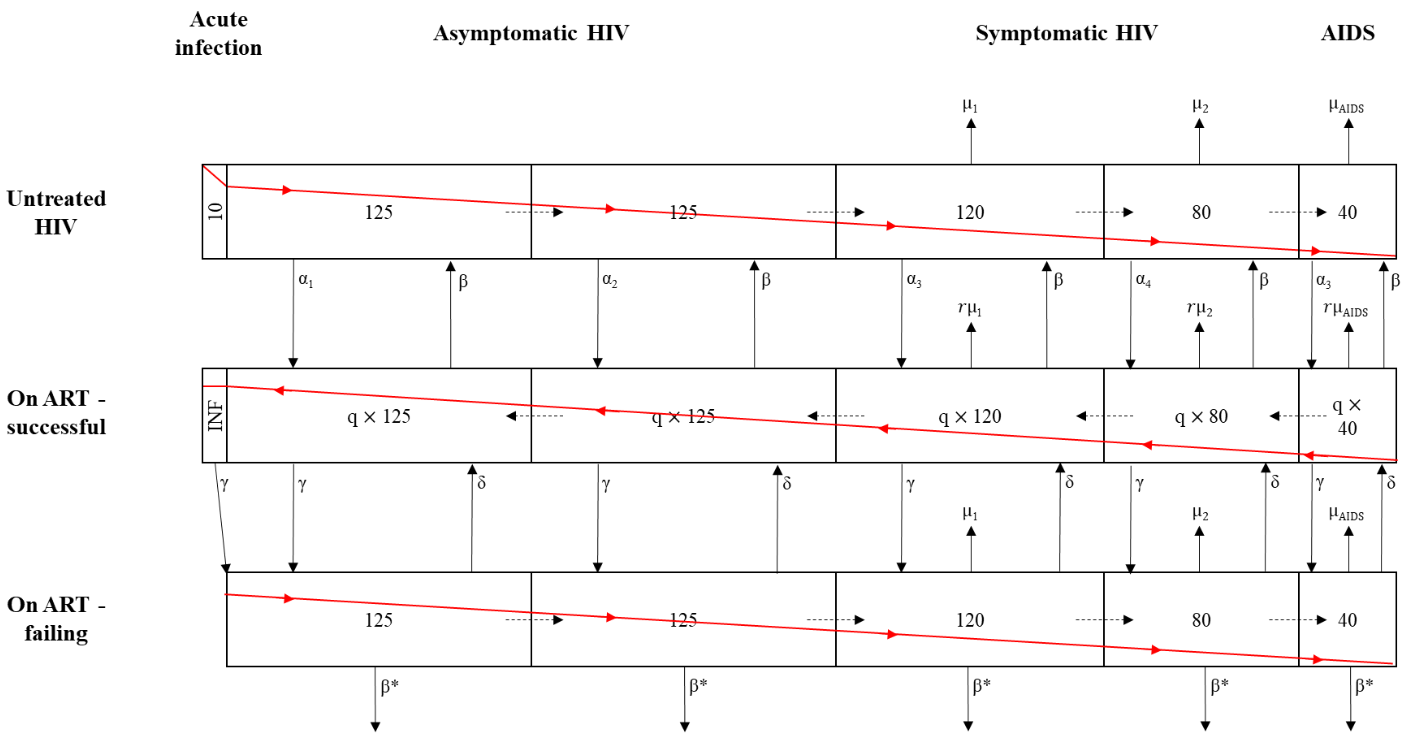

μ_1 to μ_{AIDS} = HIV-specific mortality rate (0.025, 0.05, and 1.3/year resp.)
— (red line) = relative CD4 cell count (1.0 to 0.005)
α_1 to α_5 = stage specific health seeking rates (0.1, 1.3, 2.5,3.7, and 4.9/year resp.)
β = dropout (0.013/year)
q = multiplier of CD4 cell count recovery on ART (0.5)
r = multiplier of mortality while on ART (0.5)
γ = treatment failure rate (0.115/year)
δ = re-suppression rate (0.55/year)

Figure A1. Schematic representation of stages in adjusted HIV natural history and ART processes in STDSIM. The numbers in each of the compartments represent the average duration in weeks. INF = infinite. The arrows reflecting dropout from failing ART (β*) return to the corresponding untreated HIV stages. Red lines represent the relative CD4 cell count decline (upper and lower row), from 1·0 (top of the compartments) to 0·005 of the HIV negative CD4 cell counts. The dashed arrows in the stages and the red arrows in the relative CD4 cell count lines reflect the directional flow, i.e. from left to right (disease progression) in the top and bottom row, and from right to left (health recovery) in the middle row. In the middle row, CD4 cell counts increase in the reverse order up to a maximum of 0·75 of the HIV-negative CD4 cell count. Values for each parameter are given in the legend.

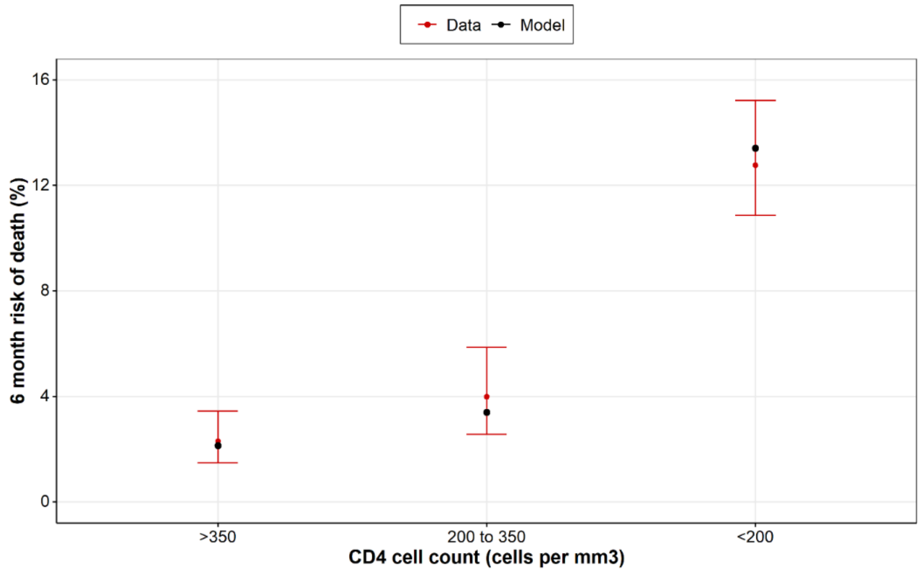

Figure A2. HIV mortality rate by CD4 cell count in untreated HIV, model compared to data. Data from Badri *et al*.[11] Mortality in the model and data reflects the combination of HIV-related mortality and other cause (i.e. background) mortality.

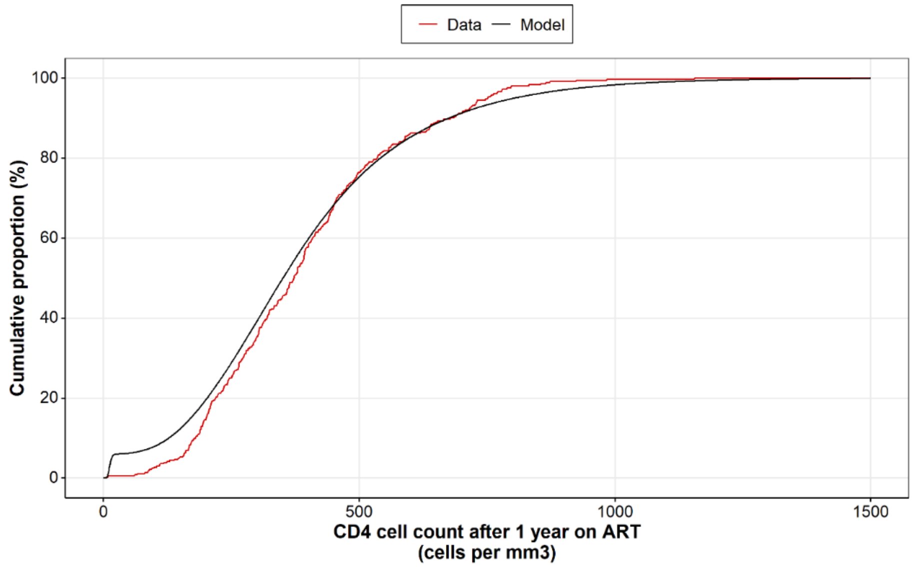

Figure A3. Cumulative distribution of CD4 cell counts after 1 year of antiretroviral therapy, model compared to data. Data from AHRI treatment program.[12]

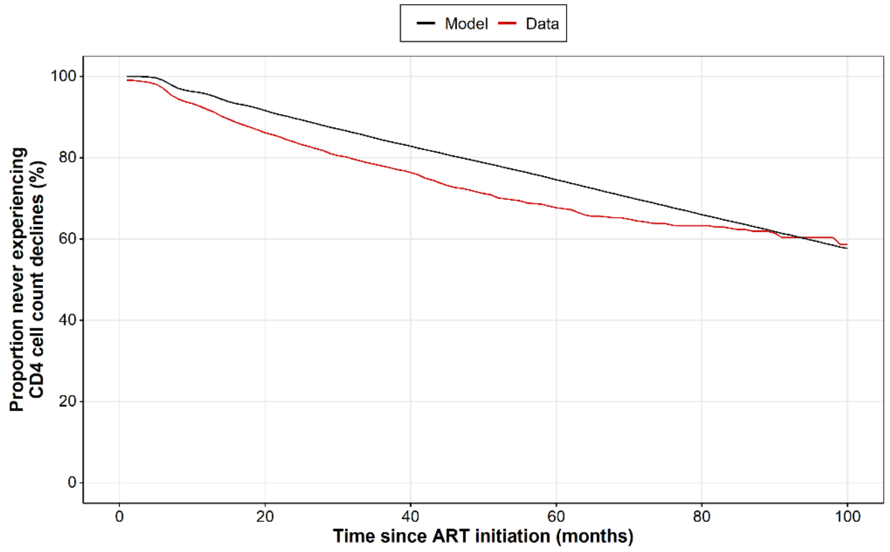

Figure A4. Proportion never failing treatment (defined as experiencing a decline in CD4 cell counts while on ART) by time on treatment, model versus data. Data from AHRI treatment program.[12]

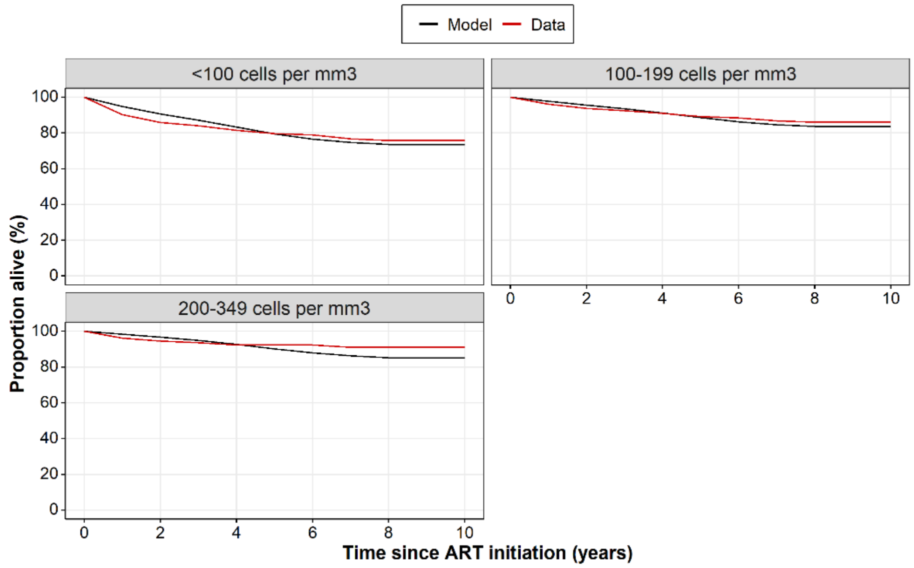

Figure A5. Mortality by time on ART and CD4 cell count at treatment initiation, model versus data. Data from AHRI treatment program.[12] Mortality in the model and data reflects the combination of HIV-related mortality and other cause (i.e. background) mortality.

1.2 Country quantifications

We used previously published quantifications for seven sub-Saharan African countries in the current analysis, as transmission risks, treatment initiation, stage durations and mortality risks were not affected by the two model adjustments.[10] In the original study, we calibrated our model to the HIV prevalence and ART uptake in the ten biggest HIV epidemics in sub-Saharan Africa (Ethiopia, Kenya, Malawi, Mozambique, Nigeria, South Africa, Tanzania, Uganda, Zambia, and Zimbabwe), using data from the period 1990 to 2014. We further used the "continued scale-up" scenario from the original study, which assumes the increases in ART access over time to continue beyond the calibration period, until full access has been reached (see Hontelez *et al*[10] for more details). For each country, we selected a set of 40 unique parameter combinations that reproduced the data according to the fitting procedure described in Hontelez *et al*.[10] We applied country specific ART guideline changes over time (i.e. to treatment eligibility at CD4 cell counts of ≤500 cells/mm^3 and to treatment eligibility for everyone living with HIV, irrespective of CD4 cell count), and compared HIV prevalence and ART coverage over the period 1990-2023 to UNAIDS data.[13] The patterns for 2015 to 2023 can thus be considered a validation of the model and its original quantifications.

Figure A6 shows country specific HIV prevalence (proportion of all people aged 15 years or older living with HIV) in the model compared to UNAIDS reported data. Every single grey line represents a unique parameter combination, and the dark grey line the median over all combinations. It is encouraging that the predicted HIV prevalence follows reported trends in HIV prevalence for each country reasonably well. In addition, the model was able to reproduce the observed ART scale-up in each country for the most part (Figure A7). For four countries (Kenya, Malawi, Zambia, and Zimbabwe) the predicted ART coverage was slightly lower compared to the UNAIDS model-produced estimates for 2022 and 2023. Finally, our model mostly matched the trends in HIV mortality rates as estimated by UNAIDS (Figure A8), which were produced using the Spectrum/AIM modeling suit.[13] For South Africa, we reduced HIV related mortality on ART (*r* multiplier in Figure A1) to 0·3 (0·5 for all other countries) to reflect relatively better healthcare for those on treatment.

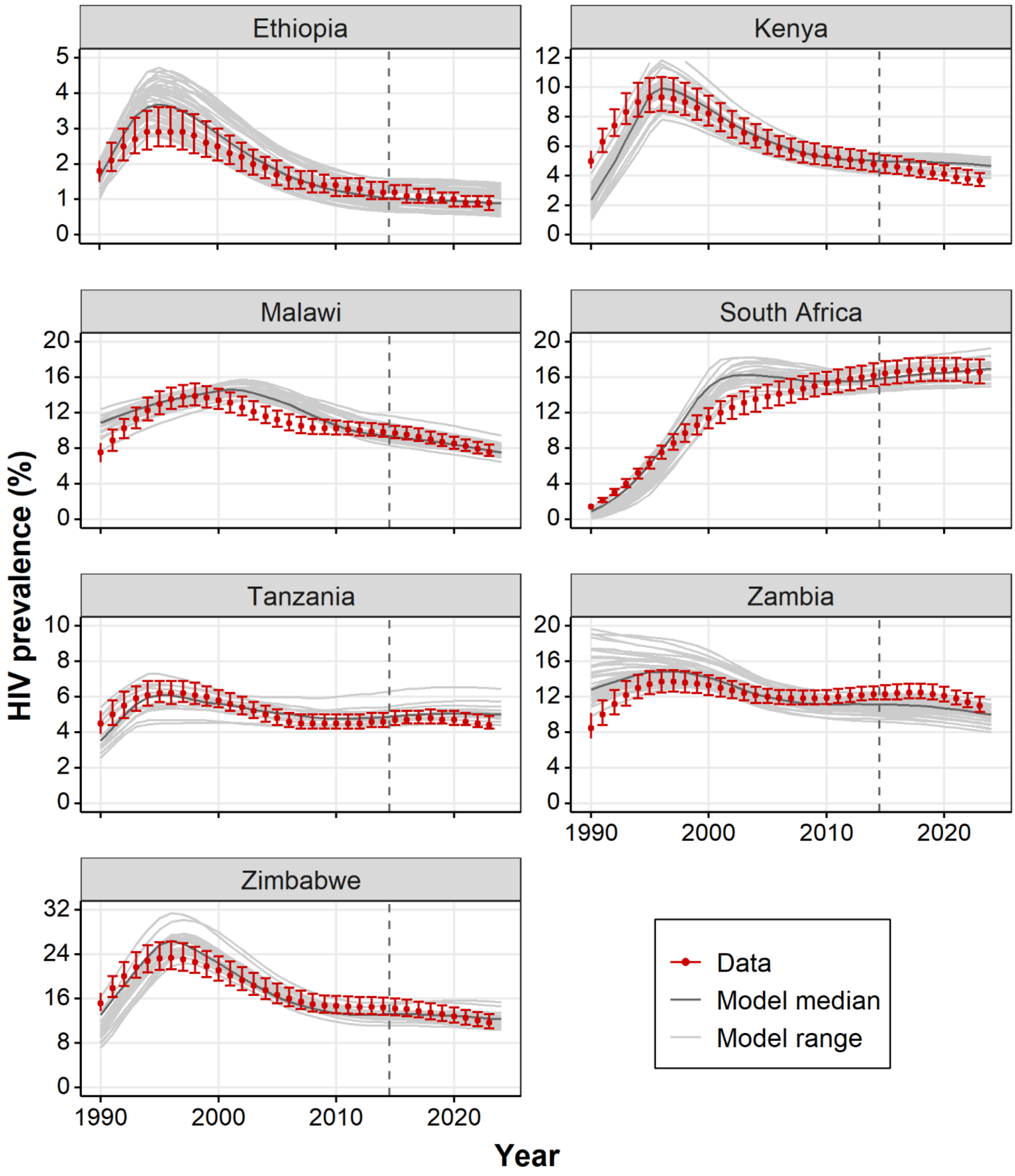

Figure A6. Modelled versus UNAIDS reported HIV prevalence in people aged 15 years or older over the period 1990 to 2023. Data derived from UNAIDS database.[13] Model predictions left of the dashed line were calibrated in Hontelez *et al*[10], and model predictions right of the dashed lines are validations against more recent data. Each light grey line represents predictions of a unique parameter combination (40 per country), dark grey line represents the median across all parameter combination predictions.

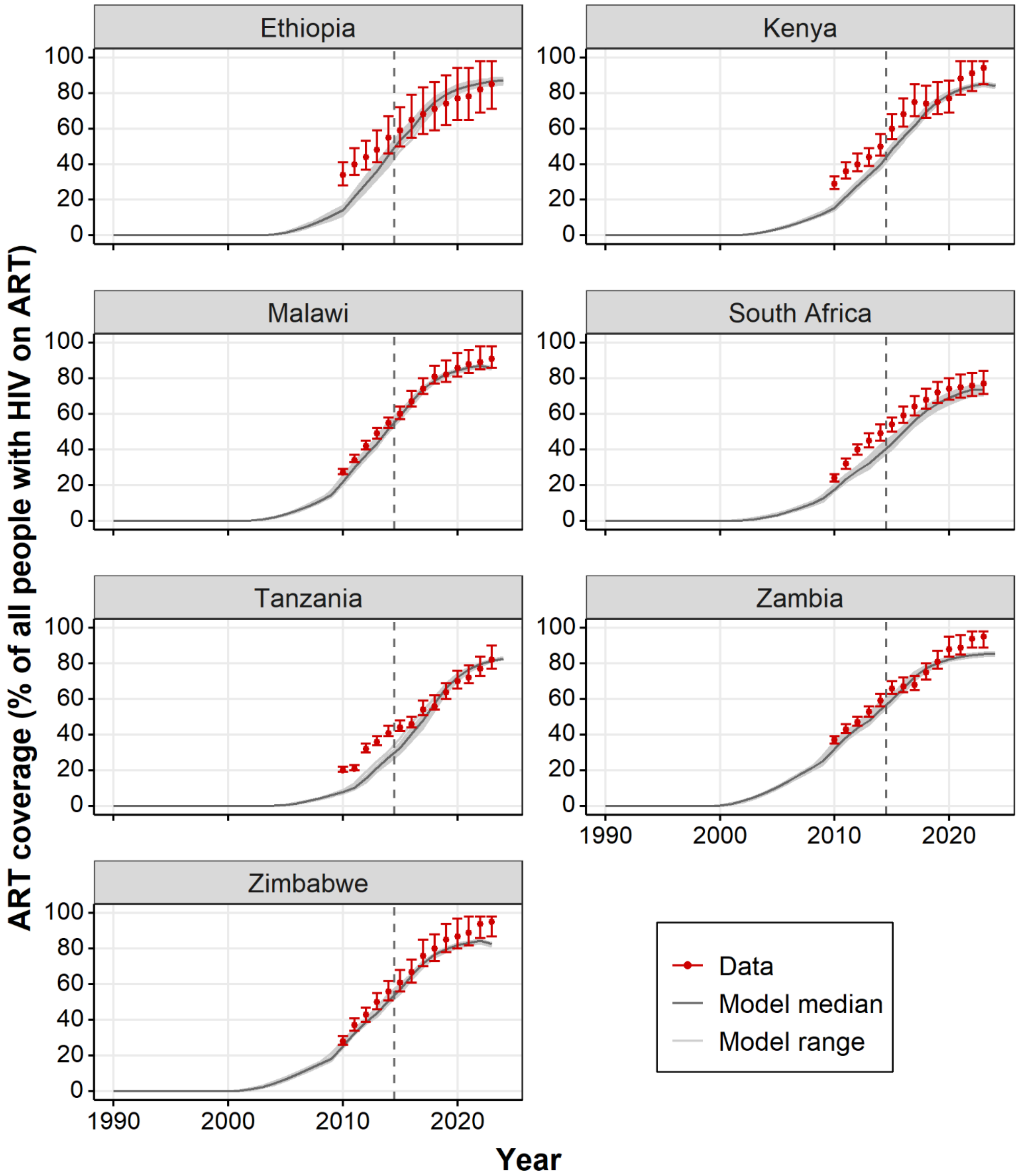

Figure A7. Modelled versus UNAIDS reported ART coverage in people aged 15 years or older living with HIV over the period 1990 to 2023. Data derived from UNAIDS database[13]. Model predictions left of the dashed line were calibrated in Hontelez *et al*[10], and model predictions right of the dashed lines are validations against more recent data. Each light grey line represents predictions of a unique parameter combination (40 per country), dark grey line represents the median across all parameter combination predictions.

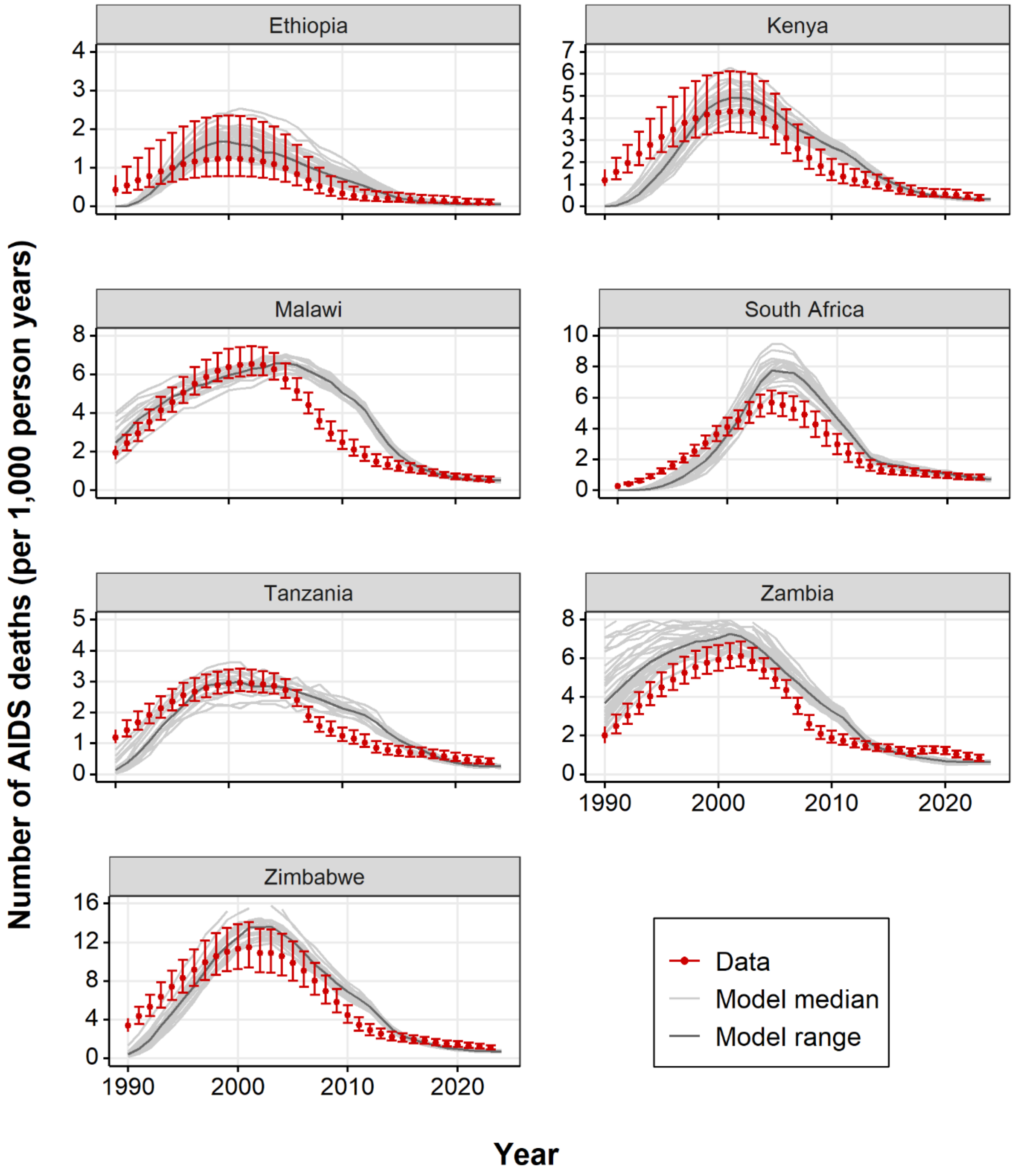

Figure A8. Modelled versus UNAIDS reported HIV related mortality in people aged 15 years or older over the period 1990 to 2023. Data derived from UNAIDS database.[13] Each light grey line represents predictions of a unique parameter combination (40 per country), dark grey line represents the median across all parameter combination predictions. The graph shows only HIV-related mortality (i.e. death due to μ_1, μ_2, and μ_{AIDS}). The model has not explicitly been fitted against these data, thus the comparisons can be considered validations.

2. Funding freeze scenarios

Table A1 gives an overview of the funding freeze scenarios, Table A2 gives the total HIV budget, and PEPFAR share within this budget, for each country in our analysis.

Table A1. Overview of *Executive order* and *Waiver* scenarios in the model.

	**Executive order – realistic**	**Executive order – proportional**	**Waiver – 8 weeks**	**Waiver – 4 weeks**
**Pause period**				
Proportion of those on treatment affected	90% of those on treatment*	Proportional to PEPFAR share in the country¶	90% of those on treatment*	90% of those on treatment*
Duration until treatment defaulting	Uniform distribution between 0 and 1 month	Uniform distribution between 0 and 1 month	Uniform distribution between 0 and 1 month	Uniform distribution between 0 and 1 month
New treatment initiations	Stopped	Stopped	Stopped	Stopped
Duration	90 days	90 days	8 weeks	4 weeks
**Re-instatement of funding**				
Average duration of return of defaulted patients	6 months	6 months	6 months	6 months
New treatment initiations	Back at pre-2025 levels after 6 months after re-instatement	Back at pre-2025 levels 6 months after re-instatement	At 25% after 8 weeks, full after 90 days	At 25% after 4 weeks, full after 90 days

¶ See Table A2 for an overview of the share of PEPFAR in each country.

*75% for Kenya and 50% for South Africa, because of the relatively modest PEPFAR contributions to those countries, see Table A2

Table A2. Overview of PEPFAR shares in total HIV spending in each country, for 2022. Source: PEPFAR Country Operational Plan (COP) 2022 of the respective country.

	Total budget (million USD)	PEPFAR contribution (million USD)	PEPFAR share	Estimated PEPFAR treatment and care share*
**Ethiopia**	278	114	41%	33%
**Kenya**	621	228	37%	33%
**Malawi**	356	171	48%	41%
**South Africa**	2,550	434	17%	12%
**Tanzania**	656	440	67%	67%
**Zambia**	530	408	77%	76%
**Zimbabwe**	417	204	49%	45%

USD = United States Dollars

*Estimated share of PEPFAR in treatment and care.

3. Country specific outcomes

This section contains additional graphs and tables of country specific outcomes regarding ART coverage (Figure A9), new HIV infections (Figure A10), HIV mortality (Figure A11). In addition, we provide a zoomed-in version of the graphs in the main manuscript showing the projected number of new HIV infections (Figure A12) and projected number of HIV deaths (Figure A13) over the period 2025 to 2027. Furthermore, an overview of country specific distributions across treatment states (i.e. ART naïve; on art < 1 year & CD4 < 350 cells/μL at initiation; on ART – other; and dropped out) before the freeze is given in Figure A14, and the distribution of excess deaths across treatment states in the *Executive order – realistic* scenario in Figure A15.

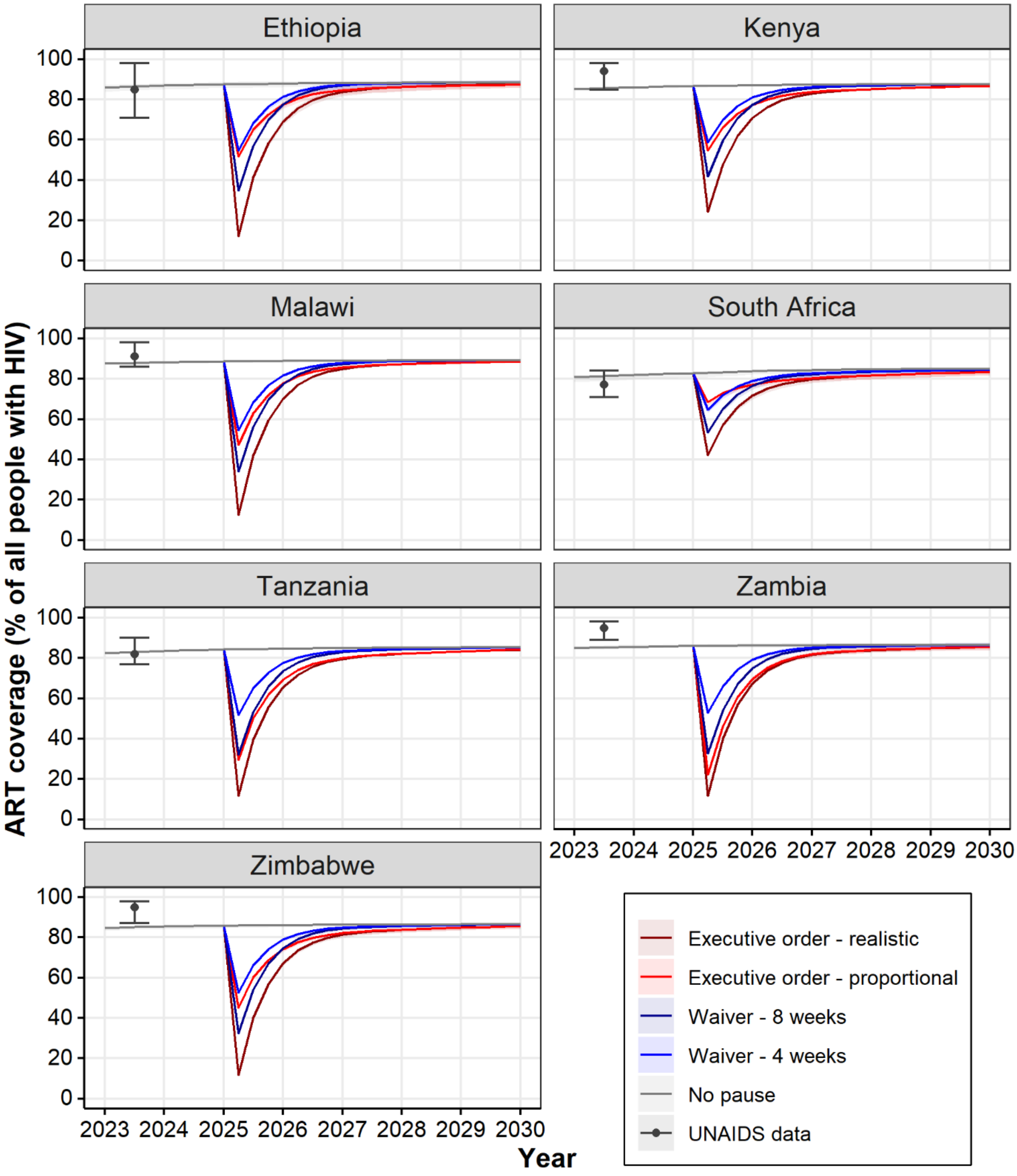

Figure A9. Predicted impact of PEPFAR funding pause scenarios on ART treatment coverage in seven countries, 2023 to 2030. Data point reflects combined UNAIDS data estimates for 2023. Solid lines reflect model means, ranges reflect 95% ranges for model predictions. Lines represent median model predictions, shadings represent 95% uncertainty intervals (UIs), with borders of the shaded area representing the 2·5% and 97·5% quantiles of model predictions.

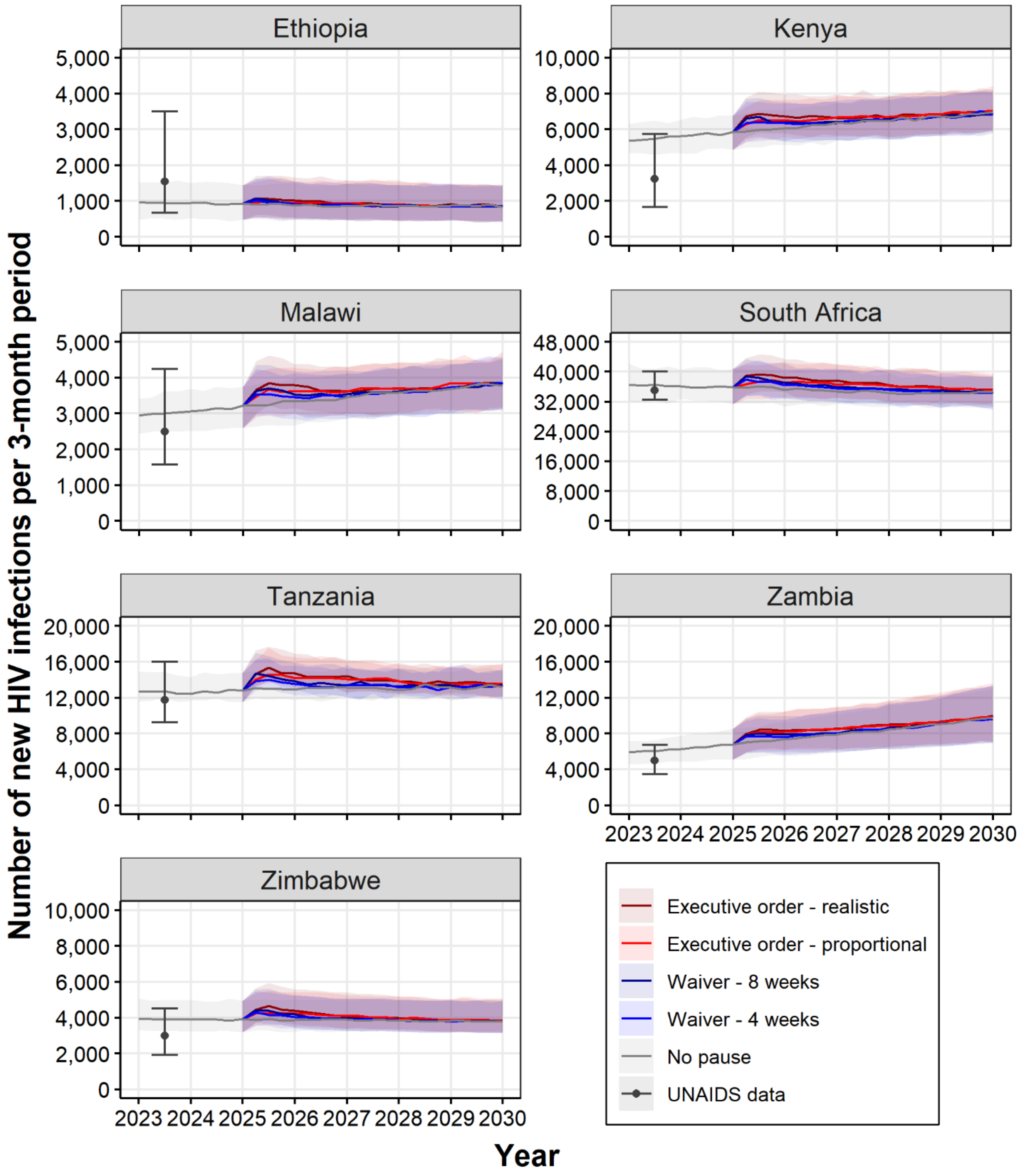

Figure A10. Predicted impact of PEPFAR funding pause scenarios on new HIV infections in seven countries, 2023 to 2030. Data point reflects combined UNAIDS data estimates for 2023. Solid lines reflect model means, ranges reflect 95% ranges for model predictions. Lines represent median model predictions, shadings represent 95% uncertainty intervals (UIs), with borders of the shaded area representing the 2·5% and 97·5% quantiles of model predictions.

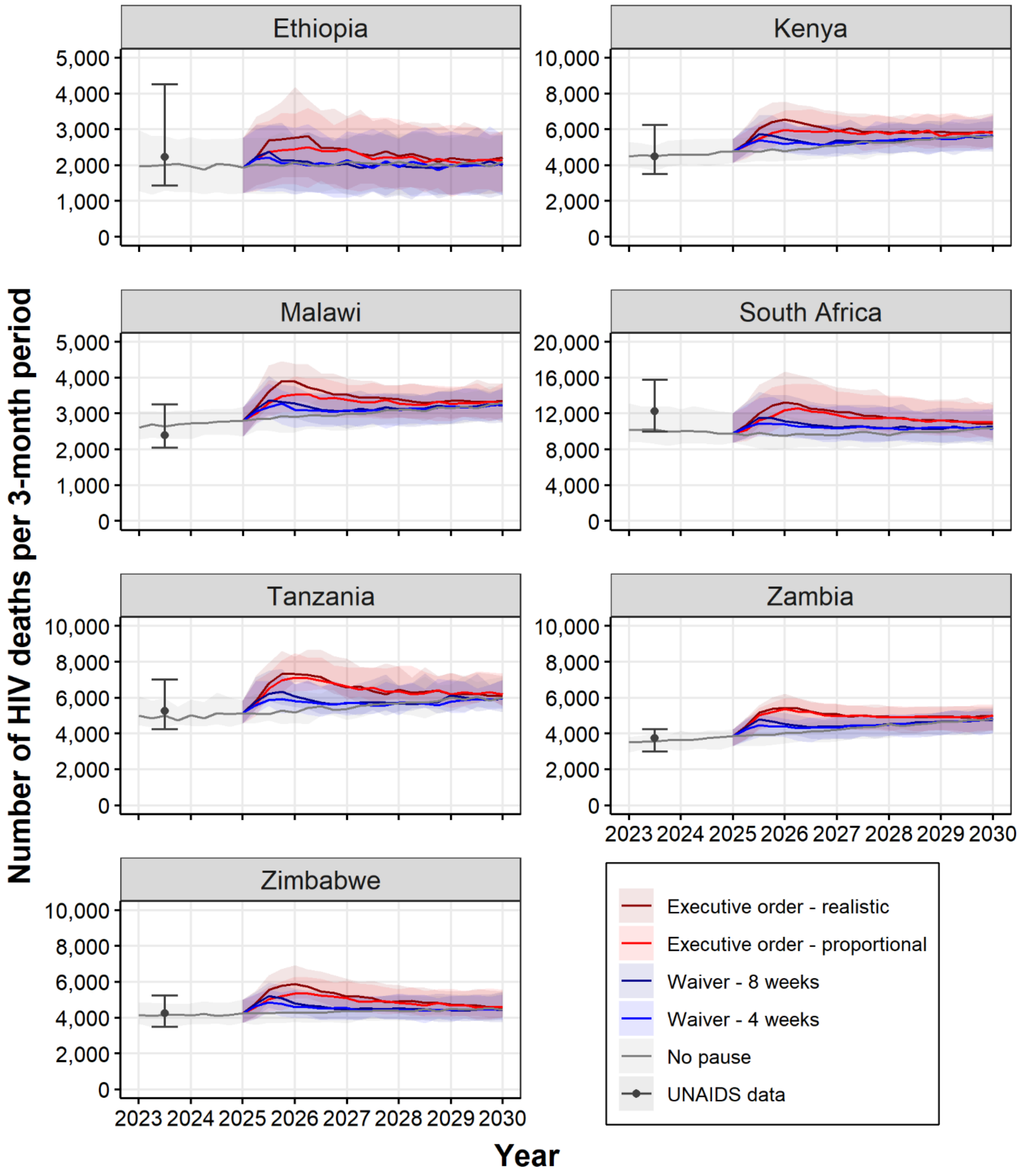

Figure A11. Predicted impact of PEPFAR funding pause scenarios on HIV deaths in seven countries, 2023 to 2030. Data point reflects combined UNAIDS data estimates for 2023. Solid lines reflect model means, ranges reflect 95% ranges for model predictions. Lines represent median model predictions, shadings represent 95% uncertainty intervals (UIs), with borders of the shaded area representing the 2·5% and 97·5% quantiles of model predictions.

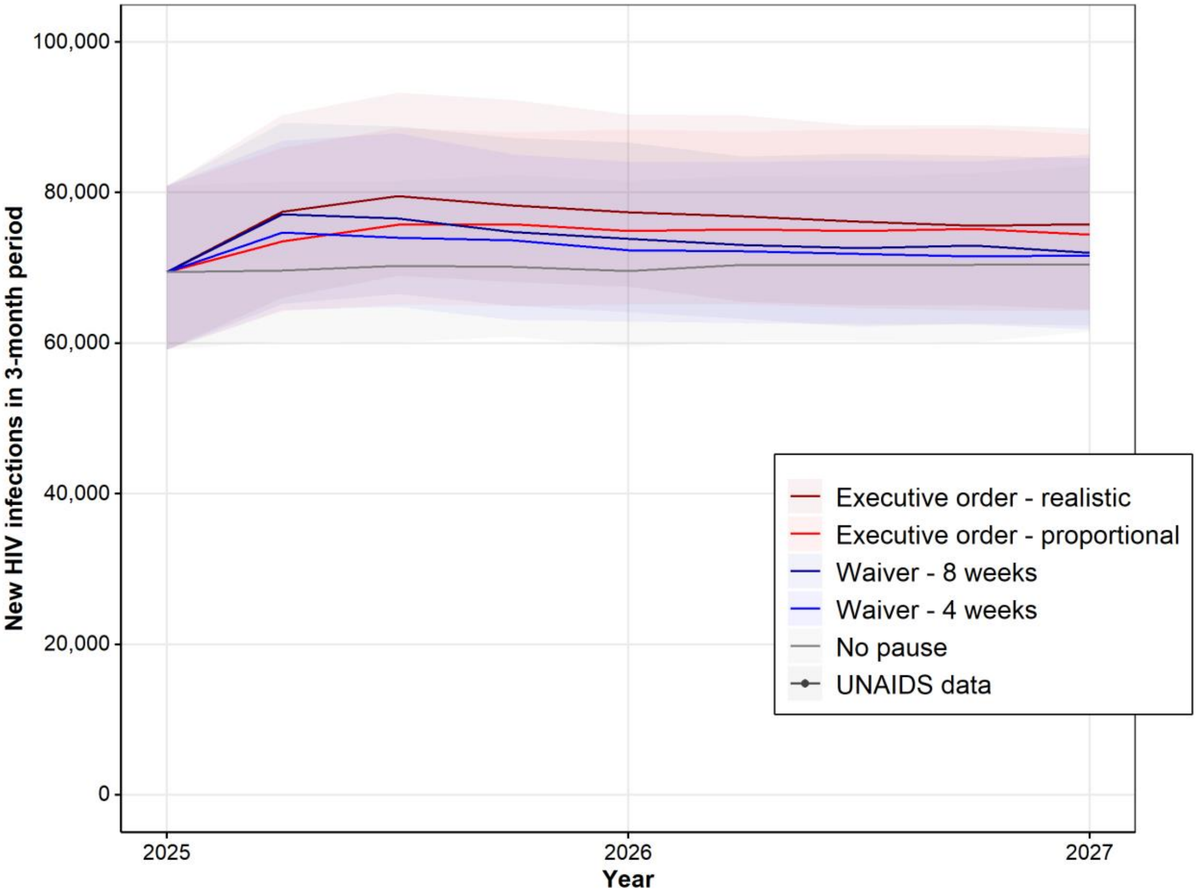

Figure A12. Projected number of new HIV infections over the period 2025 to 2027 under different PEPFAR pause scenarios. New infections are a combined total of Ethiopia, Kenya, Malawi, South Africa, Tanzania, Zambia, and Zimbabwe. Data point reflects combined UNAIDS data estimates for 2023. Lines represent median model predictions, shadings represent 95% uncertainty intervals (UIs), with borders of the shaded area representing the 2·5% and 97·5% quantiles of model predictions.

Table A3. Excess number of new HIV infections (x 1,000) due to the PEPFAR funding freeze under different scenarios. Estimates represent median model predictions, ranges represent 95% uncertainty intervals (UIs), i.e. the 2·5% and 97·5% quantiles of model predictions.

Country	Executive order - realistic	Executive order - proportional	Waiver – 8 weeks	Waiver – 4 weeks
Ethiopia	1·1<br>[0·5; 2·3]	0·9<br>[0·1; 1·9]	0·3<br>[0·1; 1·0]	0·3<br>[0·1; 0·7]
Kenya	10·2<br>[7·8; 13·2]	7·2<br>[5·4; 9·2]	4·9<br>[3·0; 6·4]	3·1<br>[1·8; 5·2]
Malawi	3·9<br>[3·2; 5·1]	2·9<br>[2·0; 3·9]	1·9<br>[1·6; 2·3]	1·2<br>[0·5; 1·7]
South Africa	51·5<br>[39·7; 57·6]	39·4<br>[32·9; 44·0]	26·2<br>[21·0 32·9]	19·9<br>[11·0; 25·6]
Tanzania	18·7<br>[16·9; 26·3]	16·3<br>[13·0; 20·2]	8·4<br>[6·3; 11·4]	5·5<br>[2·6; 9·2]
Zambia	11·0<br>[8·4; 13·8]	9·7<br>[7·3; 12·9]	4·7<br>[2·7; 6·9]	3·2<br>[1·7; 5·1]
Zimbabwe	6·2<br>[4·8; 7·8]	4·7<br>[3·5; 6·0]	3·0<br>[2·1; 3·7]	1·9<br>[1·1; 2·7]
Combine	102·6<br>[81·3; 126·0]	81·1<br>[64·4; 98·2]	49·5<br>[36·6; 64·5]	35·2<br>[18·6; 50·1]

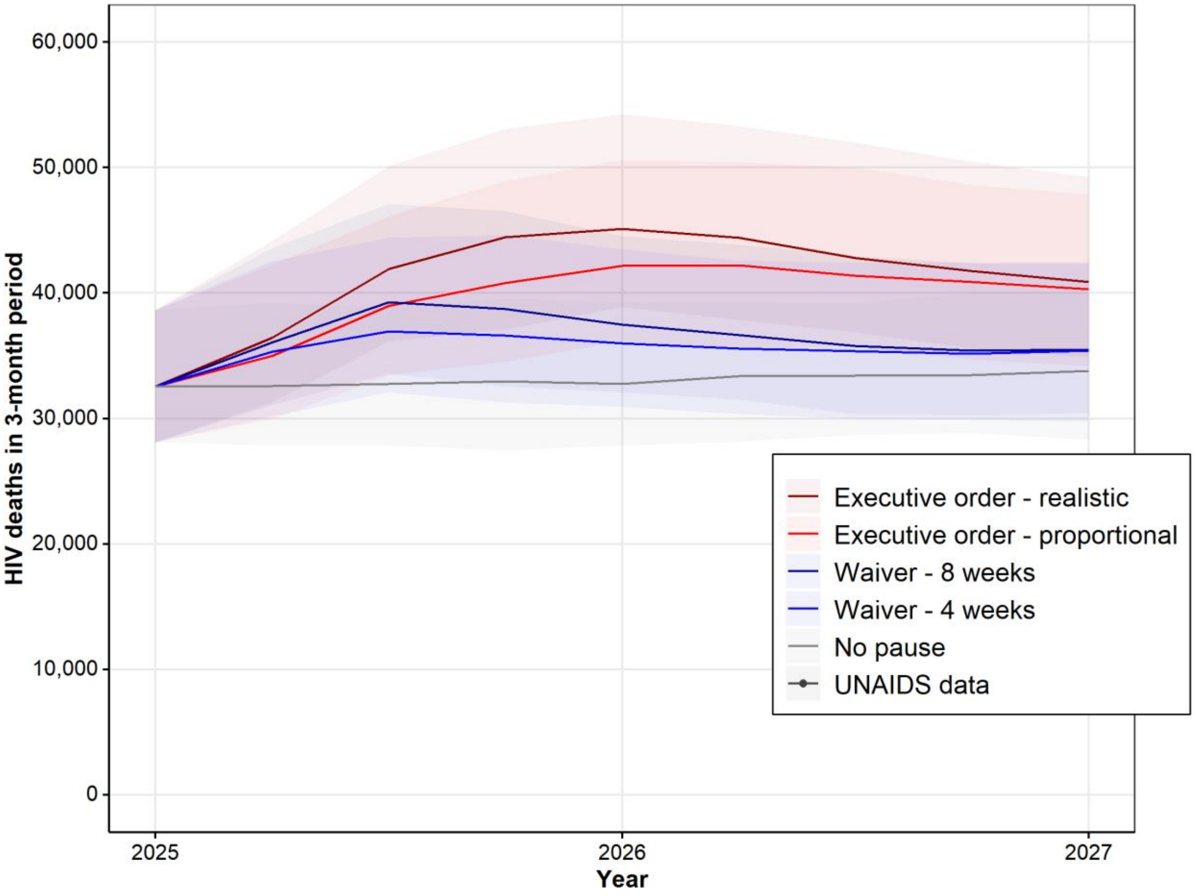

Figure A13. Projected number of HIV deaths over the period 2025 to 2027 under different PEPFAR pause scenarios. Results contain the following countries: Ethiopia, Kenya, Malawi, South Africa, Tanzania, Zambia, and Zimbabwe. Data point reflects combined UNAIDS data estimates for 2023. Lines represent median model predictions, shadings represent 95% uncertainty intervals (UIs), with borders of the shaded area representing the 2·5% and 97·5% quantiles of model predictions.

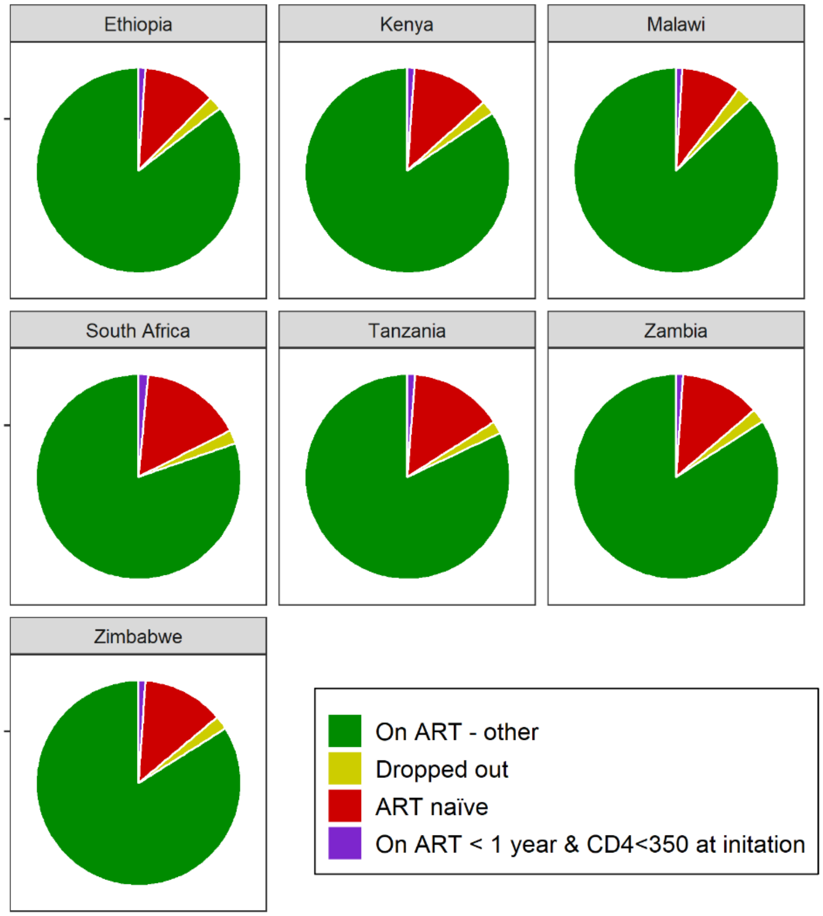

Figure A14. Distribution of people living with HIV across ART treatment categories before the freeze.

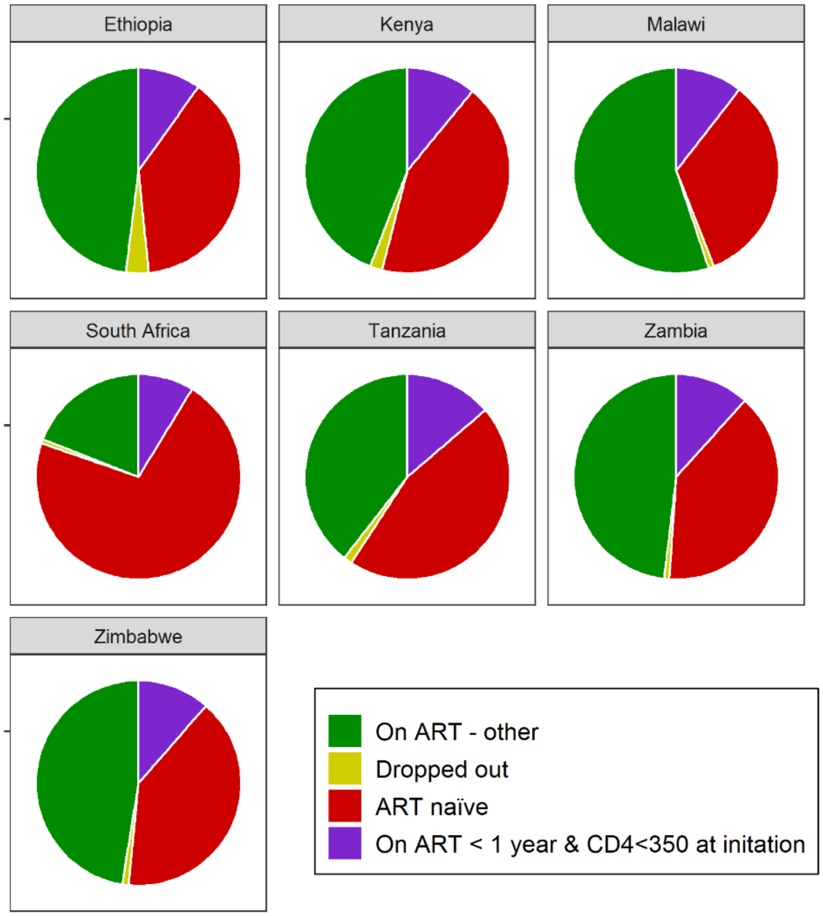

Figure A15. Distribution of excess deaths across ART treatment categories at the start of the freeze for the *Executive order – realistic* scenario.

4. References

1. Ploeg CPBV der, Vliet CV, Vlas SJD, et al. STDSIM: A Microsimulation Model for Decision Support in STD Control. *Interfaces*. 1998;28(3):84-100.

2. Freeman EE, White RG, Bakker R, et al. Population-level effect of potential HSV2 prophylactic vaccines on HIV incidence in sub-Saharan Africa. *Vaccine*. 2009;27(6):940-946. doi:10.1016/j.vaccine.2008.11.074

3. Korenromp EL, Bakker R, De Vlas SJ, Robinson NJ, Hayes R, Habbema JDF. Can behavior change explain increases in the proportion of genital ulcers attributable to herpes in sub-Saharan Africa? A simulation modeling study. *Sex Transm Dis*. 2002;29(4):228-238. doi:10.1097/00007435-200204000-00008

4. Korenromp EL, Bakker R, Gray R, Wawer MJ, Serwadda D, Habbema JDF. The effect of HIV, behavioural change, and STD syndromic management on STD epidemiology in sub-Saharan Africa: simulations of Uganda. *Sex Transm Infect*. 2002;78 Suppl 1(Suppl 1):i55-63. doi:10.1136/sti.78.suppl_1.i55

5. Hontelez JAC, de Vlas SJ, Tanser F, et al. The impact of the new WHO antiretroviral treatment guidelines on HIV epidemic dynamics and cost in South Africa. *PLoS One*. 2011;6(7):e21919. doi:10.1371/journal.pone.0021919

6. Hontelez JAC, Lurie MN, Newell ML, et al. Ageing with HIV in South Africa. *AIDS*. 2011;25(13):1665-1667. doi:10.1097/QAD.0b013e32834982ea

7. Hontelez JA, Nagelkerke N, Bärnighausen T, et al. The potential impact of RV144-like vaccines in rural South Africa: a study using the STDSIM microsimulation model. *Vaccine*. 2011;29(36):6100-6106. doi:10.1016/j.vaccine.2011.06.059

8. Hontelez JA, de Vlas SJ, Baltussen R, et al. The impact of antiretroviral treatment on the age composition of the HIV epidemic in sub-Saharan Africa. *AIDS*. 2012;26(0):10.1097/QAD.0b013e3283558526. doi:10.1097/QAD.0b013e3283558526

9. Hontelez JAC, Lurie MN, Bärnighausen T, et al. Elimination of HIV in South Africa through Expanded Access to Antiretroviral Therapy: A Model Comparison Study. *PLoS Med*. 2013;10(10):e1001534. doi:10.1371/journal.pmed.1001534

10. Hontelez JAC, Chang AY, Ogbuoji O, de Vlas SJ, Bärnighausen T, Atun R. Changing HIV treatment eligibility under health system constraints in sub-Saharan Africa: investment needs, population health gains, and cost-effectiveness. *AIDS*. 2016;30(15):2341-2350. doi:10.1097/QAD.0000000000001190

11. Badri M, Lawn SD, Wood R. Short-term risk of AIDS or death in people infected with HIV-1 before antiretroviral therapy in South Africa: a longitudinal study. *Lancet*. 2006;368(9543):1254-1259. doi:10.1016/S0140-6736(06)69117-4

12. AHRI data respository. Accessed February 20, 2025. https://data.ahri.org/index.php/home

13. AIDSinfo | UNAIDS. Accessed February 8, 2025. https://aidsinfo.unaids.org/
